# Characterization of Breast Cancer Preclinical Models Reveals a Specific Pattern of Macrophage Polarization

**DOI:** 10.1371/journal.pone.0157670

**Published:** 2016-07-07

**Authors:** David Vallerand, Gérald Massonnet, Fatima Kébir, David Gentien, Zofia Maciorowski, Pierre De la Grange, Brigitte Sigal-Zafrani, Marion Richardson, Sandrine Humbert, Aurélie Thuleau, Franck Assayag, Ludmilla de Plater, André Nicolas, Suzy Scholl, Elisabetta Marangoni, Stefan Weigand, Sergio Roman-Roman, Ariel Savina, Didier Decaudin

**Affiliations:** 1 Translational Research Department, Laboratory of Preclinical Investigation, Institut Curie, PSL University, Paris, France; 2 Institut Roche, Boulogne-Billancourt, France; 3 Department of Pathology, Institut Curie, Paris, France; 4 Platform of Molecular Biology Facilities, Institut Curie, PSL University, Paris, France; 5 Flow Cytometry Core Facility, Institut Curie, PSL University, Paris, France; 6 GenoSplice Technology, Institut Universitaire d’Hématologie, Paris, France; 7 Inserm, U830, Institut Curie, PSL University, Paris, France; 8 CNRS UMR3306, INSERM U1005, Institut Curie, PSL University, Orsay, France; 9 Department of Medical Oncology, Institut Curie, Institut Curie, Paris, France; 10 Roche Diagnostics GmbH, Penzberg, Germany; 11 Translational Research Department, Institut Curie, PSL University, Paris, France; University of Tennessee Health Science Center, UNITED STATES

## Abstract

Drug discovery efforts have focused on the tumor microenvironment in recent years. However, few studies have characterized the stroma component in patient-derived xenografts (PDXs) and genetically engineered mouse models (GEMs). In this study, we characterized the stroma in various models of breast cancer tumors in mice. We performed transcriptomic and flow cytometry analyses on murine populations for a series of 25 PDXs and the two most commonly used GEMs (MMTV-PyMT and MMTV-erBb2). We sorted macrophages from five models. We then profiled gene expression in these cells, which were also subjected to flow cytometry for phenotypic characterization. Hematopoietic cell composition, mostly macrophages and granulocytes, differed between tumors. Macrophages had a specific polarization phenotype related to their M1/M2 classification and associated with the expression of genes involved in the recruitment, invasion and metastasis processes. The heterogeneity of the stroma component of the models studied suggests that tumor cells modify their microenvironment to satisfy their needs. Our observations suggest that such models are of relevance for preclinical studies.

## Introduction

Most preclinical studies on breast cancer (BC) to date have focused on the carcinogenesis and molecular mechanisms of this disease, including specific genetic and epigenetic alterations [1]. Cancer cells, whatever their origin, must establish a close relationship with their environment for growth and dedifferentiation or protection from immune surveillance[2]. The tumor-associated stroma plays an essential role in tumor development and maintenance [3]. Various types of stromal cells, including fibroblasts, hematopoietic and endothelial cells, infiltrate the tumor, affecting all steps in cancer development—cell growth, invasion, neoangiogenesis, metastasis—and treatment sensitivity [4–6]. It has been suggested that tumor-stroma interactions could be targeted for the treatment of human cancers [7, 8].

Preclinical investigations are an essential step in the selection of new anticancer molecules and the choice of an appropriate preclinical tumor model is crucial. The two most widely used types of preclinical cancer model are patient-derived xenografts (PDXs) and genetically engineered mouse models (GEMs)[9]. PDXs mimic the significant heterogeneity of human cancers[10–13], and can be used to evaluate combined therapies[14] through highly standardized *in vivo* pharmacological assays. GEMs are particularly relevant because they involve the spontaneous development of organ-specific tumors in an immunocompetent environment in the context of specific driver mutations, potentially providing insight into the mode of action of the underlying genetic mechanisms in addition to mimicking human pathophysiology[15].

All studies on PDXs, including human breast cancer xenografts (HBC-x) in particular, have focused on tumor cell features, such as morphology and genetic mutations, genomic and gene expression profiles. Few data are available for the tumor-associated stroma. Recent studies have shown that stromal abundance, necrotic and inflammatory areas are very similar in the tumors of patients and in the corresponding xenografts [10, 11, 16–20]. It has been shown that the human-derived stroma of PDXs is rapidly replaced by mouse-derived stroma [16, 21]. However, there has been no comparison of tumor stroma between different PDXs. The first goal of this study was to investigate the heterogeneity of stromal features in breast cancer PDXs. The second goal was to evaluate the impact on stromal components of the subcutaneous transplantation of primary spontaneous GEM tumors into immunodeficient mice.

## Materials and Methods

### Ethics statement

All patients gave verbal informed consent for experimental studies on the tumor tissue remaining after histological and cytogenetic analyses, during their first consultation at the Institut Curie. The PDXs were established after this consent had been given. All patient information was rendered anonymous. PDXs were established with the approval of the ethics committee of the Institut Curie. In accordance with French regulations and the recommendations of the ethics committee of the Institut Curie, no written consent from patients was required to obtain residual tumor tissues. This procedure was approved by the relevant ethics committees, and all the research was carried out in France. Studies were performed in accordance with the recommendations of the French Ethics Committee and under the supervision of investigators with the appropriate authorizations. The experimental protocol and animal housing complied with the institutional guidelines established by the French Ethics Committee (Agreement C75-05–18, France). The Institut Curie ethics committee approved this project and the use of mice for these studies. All surgery was performed on animals anesthetized with xylazine/ketamine, and every possible effort was made to minimize suffering. Animals were killed humanely, by cervical dislocation. All animals were monitored twice weekly, or daily if unexpected deaths occurred. Tumor size was measured twice weekly from the start of treatment.

### Preclinical models

Several preclinical GEM and PDX models of mouse and human breast cancer were used.

The GEMs models studied were breast tumors arising spontaneously in transgenic mice, *Polyoma Middle-T* antigen (PyMT) mice [22] and tumors induced by the mouse mammary tumor virus (MMTV)-ErbB2 [23]. Secondary tumors (BC-PyMT and BC-ErbB2) were obtained by the subcutaneous transplantation of spontaneous tumors into the interscapular region of Swiss *nude* mice. Five- to six-week-old female FVB/N-Tg(MMTV-PyMT)634Mul (PyMT) hemizygous transgenic mice were provided by Jackson Laboratories (Bar Harbor, Maine). Transgenic five- to six-week-old female MMTV-ErbB2 mice [24] were generously provided by Dr. Sandrine Humbert (CNRS UMR 3306 / INSERM U1005, Institut Curie, France).

The human breast cancer models were PDXs developed in our laboratory [11, 17–19, 25]. They were established from human breast cancer specimens removed with the informed consent of the patients, during surgery. These specimens were then transplanted into nude mice. All *in vivo* experiments were performed in accordance with the United Kingdom Co-ordinating Committee on Cancer Research animal ethics guidelines [26] and the institutional guidelines of the French Ethics Committee (Agreement B75-05–18, France).

### Immunohistochemistry analysis

Nine PDXs were included in the stromal study (**S1 Table**). RT-PCR analyses showed that four of the breast cancers from which these PDXs were established were triple-negative (HBCx-4B/8/12A/24), three were luminal B tumors (HBCx-3/22/34) and two were human epidermal growth factor receptor 2 (Her2)-positive breast cancers (HBCx-13B/41). We evaluated tissue morphology and fibrosis on hematoxylin-eosin-safranin (HES) and Masson’s trichrome-stained sections, respectively. The tumors were stained with stroma-specific antibodies, for the quantification of endothelial cells (CD31), and myofibroblasts (α-SMA).

Xenografts were fixed with formaldehyde-acetic acid-alcohol (A.F.A, Labonord SAS) and embedded in paraffin. We cut 4 μm sections, which were stained with hematoxylin-eosin-safranin (HES) in accordance with standard histological procedures using a Leica ST 5020 multistainer. In brief, tissue sections were dehydrated, and then heated in sodium citrate buffer or ethylenediaminetetraacetic acid (EDTA) buffer. The sections were incubated with 3% hydrogen peroxide to block endogenous peroxidase activity. They were then blocked by incubation with 2% normal serum from the same host used to generate the secondary antibodies, at 25°C for 10 minutes, and incubated with the primary antibodies at room temperature for 1 hour. The primary antibodies are shown in **S2 Table**. Sections were incubated with secondary antibodies for 30 minutes at room temperature and antibody binding was detected with a peroxidase-based detection kit (Vector Laboratories), as previously described. For each preclinical model, we studied at least three different tumors. We analyzed the images for three randomly selected sections per tumor. All sections were analyzed by a pathologist.

### Tissue dissociation

For flow cytometry, tumor and tumor-associated stromal cells were obtained by subjecting 1500 mm^3^ tumor specimens to a tissue dissociation procedure optimized for our tumors, as described by Petit *et al*. [27]. Trypan blue exclusion showed that 60–70% of the cells were viable.

### Flow cytometry

We analyzed 21 PDXs in the stromal study (**S1 Table**). The single-cell suspensions generated by tissue dissociation were incubated with rat anti-mouse CD16/CD32 antibody (BD Biosciences) at a concentration of 1 μg/10^6^ cells in 100 μl phosphate-buffered saline (PBS)/ 5% fetal calf serum (FCS), to block nonspecific binding. The cells were then stained with the appropriate monoclonal antibodies (listed in **S3 Table)**. The stained cells were washed twice with PBS/FCS. We added 2 ng/ml DAPI (4',6'-diamidino-2-phenylindole, Invitrogen) to distinguish between live and dead cells. Data were acquired with a standard LSRII flow cytometer (BD Biosciences) equipped with 20 mW 488 nm, 20 mW 633 nm and 25 mW 406 nm lasers. We used the following filters for the measurement of fluorescence emission: 530/30 for FITC or AF488, 575/26 for PE, 610/20 for PE-TX Red, 660/20 for PE-Cy5, 695/40 for PerCP-Cy5.5 or PerCP-eFluor710, 780/60 for APC-Cy7, 660/30 for APC, 730/45 for AF700, 780/60 for APC-Cy7 and 450/50 for DAPI. Data were analyzed with FlowJo Software (Treestar, Ashland OR). For each model, we studied at least three tumors.

### Cell sorting for gene expression analysis

Macrophage/monocyte-like cells (EpCAM^-^CD45^+^F4/80^+^) were sorted on a FACS Vantage DiVa (BD Biosciences) equipped with 200 mW 488nm, 35 mW 633 nm and 150 mW 365 nm lasers. We used the following filters for the measurement of fluorescence emission: 610/20 for PE-TX Red, 695/40 for PerCP-Cy5.5, 660/20 for APC and 450/20 for DAPI. The median purity of the sorted cells was 90% (range: 79.1%-96.2%) (**S7 and S8 Figs**).

### RNA extraction and array hybridization

Total RNA was isolated from the sorted fractions with the RNAeasy Plus Micro kit (Qiagen, Inc.). RNA quality was assessed by capillary electrophoresis (Bioanalyzer, Agilent, Inc.) with RNA 6000 Pico LabChip kits, which can be used for quantification and to assess the integrity of the samples. The mean RNA integrity number (RIN) was 8.2 (range: 6.9 to 9.3) for the 21 mouse macrophage-like sorted fractions. The RNA yield of the sorted cells was 1.6 pg per cell (range 0.2–6.2).

Microarray profiling was performed with 500 pg of total RNA. The NugenOvationWTA-SLv2 protocol (Nugen, Inc.) was used to prepare the targets for microarray hybridization. The SPIA amplification method (Nugen) produced a mean of 3.5 μg cDNA (range: 2.6–4.9 μg). We hybridized 2.5 μg cDNA to Affymetrix Mouse Gene ST 1.1 arrays, in accordance with Affymetrix recommendations. Raw data were extracted with the Affymetrix Expression Console (Affymetrix, Inc.). A universal mouse reference RNA (Stratagene, Inc.) was added during the synthesis and hybridization steps, to validate amplification and hybridization.

### Microarray data analysis

Quality control was performed on the Affymetrix Mouse Gene 1.1 array datasets with Expression console software (Affymetrix). Further analysis and visualization were performed with EASANA^®^ (GenoSplice technology, www.genosplice.com), with GenoSplice FAST DB^®^ annotations [28, 29]. Gene array data were normalized by quantile normalization. Background corrections were made with antigenomic probes selected as previously described [30]. Only probes targeting exons annotated from FAST DB^®^ transcripts were selected, to ensure that we focused on well-annotated genes for which mRNA sequences were present in public databases [28, 29]. Low-quality probes (*e*.*g*., probes labeled by Affymetrix as ‘cross-hybridizing’) and probes with a low signal intensity (relative to antigenomic background probes with the same GC content) were removed from the analysis. Only probes with a detection above background (DABG) *P* value ≤ 0.05 in at least half the arrays were considered for statistical analysis [30]. Only genes expressed in at least one of the conditions compared were analyzed. Expression was defined as a DABG *P*-value ≤0.05 for at least half the gene probes. An unpaired Student’s *t-*test was used to compare gene expression intensities between the various biological replicates. Genes were considered to display significant regulation of we obtained a fold-change value ≥ 1.5 and a *P* value ≤ 0.05. The data have been deposited in GEO GSE80410.

The distance from the gene signal in a given sample to the corresponding mean for all samples was calculated for each regulated gene. The corresponding values were displayed and clustered with MeV4.6.2 from the Institute of Genome Research, using Pearson correlation and average linkage clustering or dChip Software [31]. Significant GO terms were retrieved using the Database for Annotation, Visualization and Integrated Discovery (DAVID)[32] on the results for all genes, and on those for up- and downregulated genes considered separately, using DAVID EASE score ≤ 0.1. The EASE score is a modified Fisher’s exact test that “penalizes” the classical Fisher’s exact test *p*-value by subtracting 1 from the number of counts of positive agreement.

### Statistical analyses

All values are means ± SEM, with the number of animals indicated. Data were analyzed by appropriate parametric or nonparametric statistical methods, as indicated in the figure legends and tables, with Statistica^Ⓡ^ software (Stasoft), except for gene expression analysis. A *p*-value less than 0.05 was considered significant. The flow cytometry plots are representative of the replicated experiments.

## Results

### Histological characterization of the stroma of PDX tumor models

**Fig 1:**
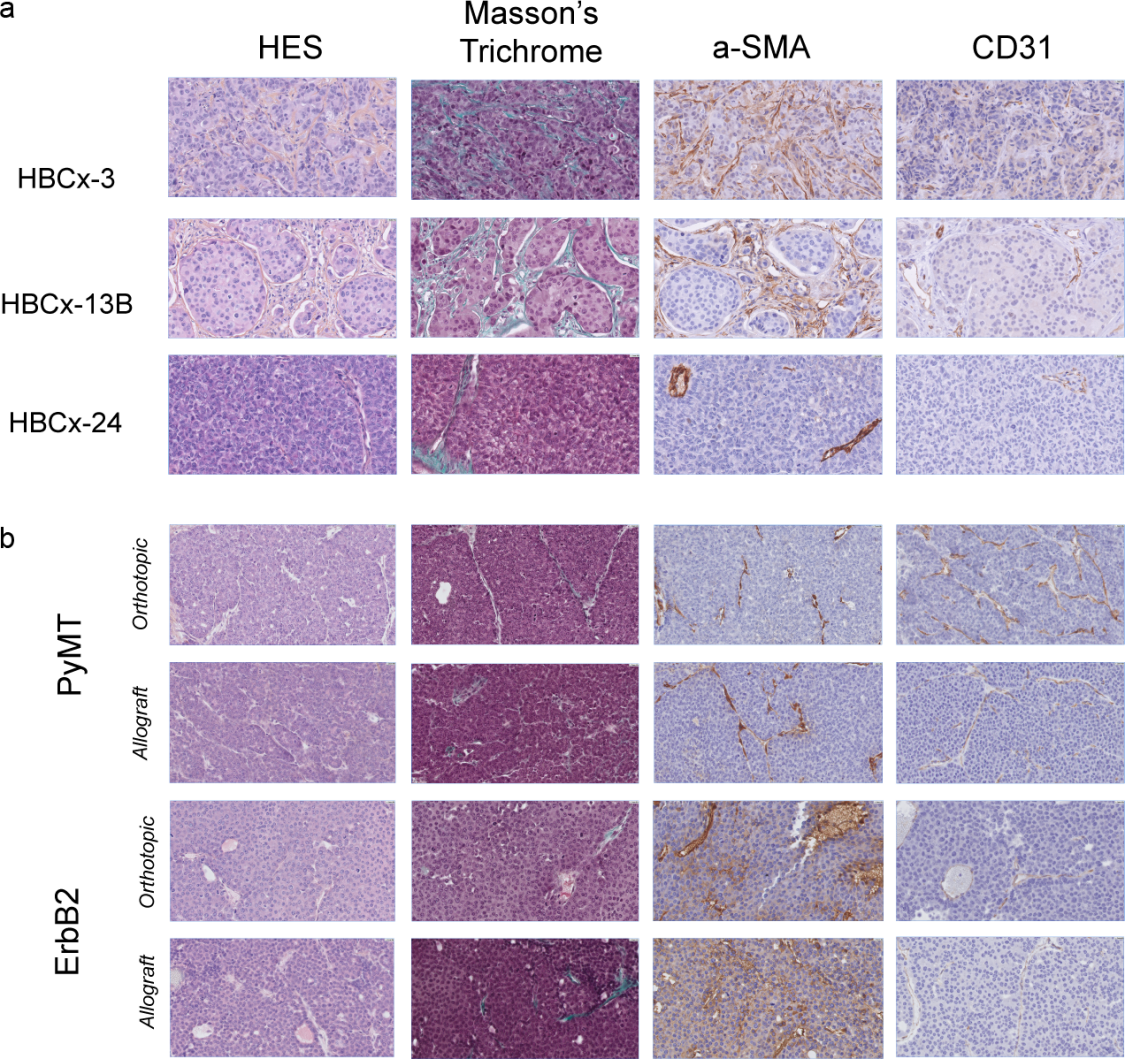
Histology and immunohistochemistry of myofibroblasts and endothelial cells in human and mouse breast cancer tumors. Slides stained with hematoxylin, eosin and safranin (HES) and Masson’s trichrome, and stained for α-SMA and CD31: (**a**) human PDXs and (**b**) murine PyMT and ErbB2 tumors (original magnification: 400x).

**Fig 2:**
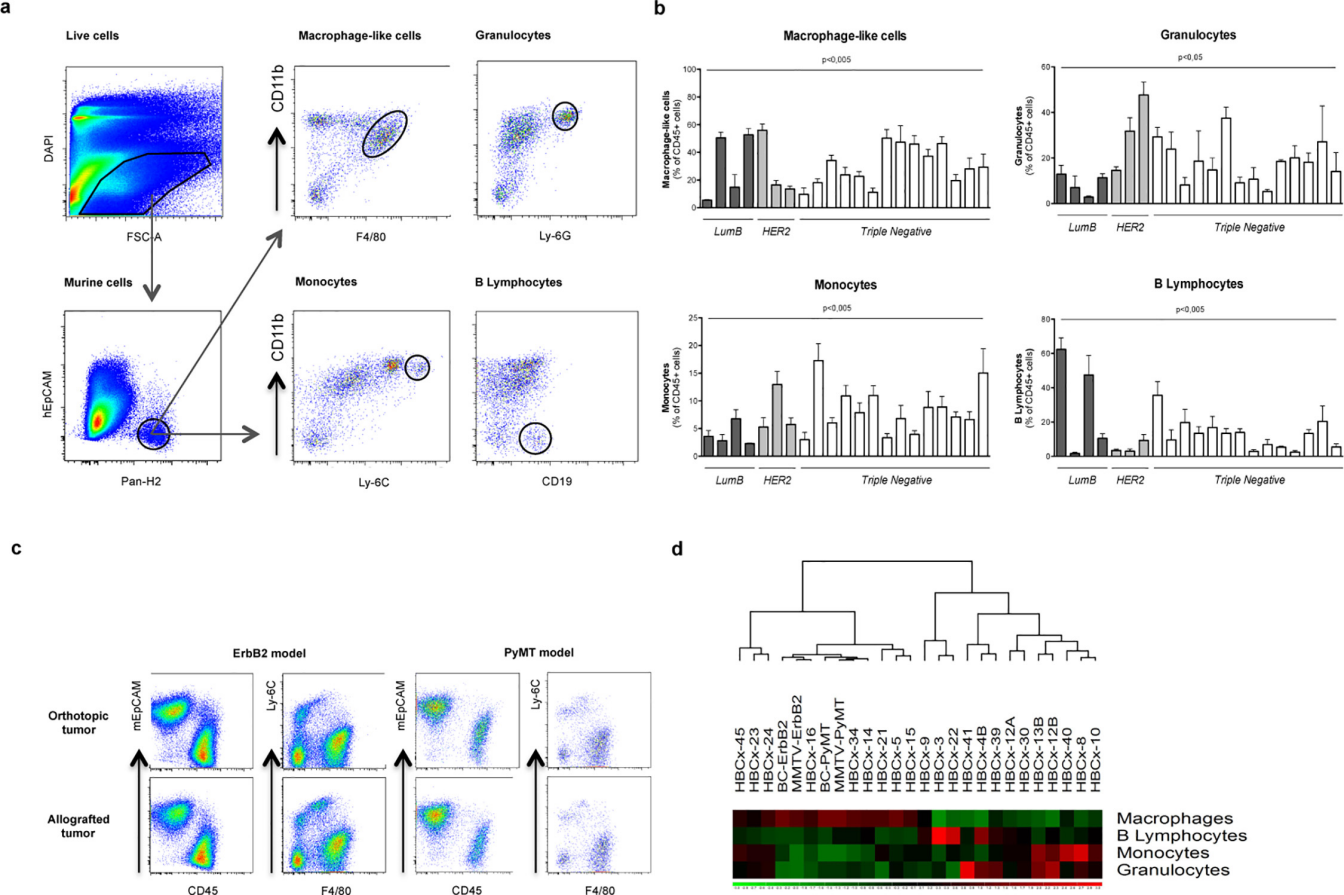
Heterogeneity of mouse-derived stroma in breast cancer tumors. (**a**) Flow cytometry analysis of tumor-infiltrating hematopoietic stromal cells after dissociation of the HBCx-9 tumor graft and staining with anti-EpCAM, CD45, F4/80, Ly-6G, Ly-6C and CD19 antibodies. Viable murine macrophage-like cells (DAPI^-^EpCAM^-^CD45^+^CD11b^+^F4/80^+^), monocytes (DAPI^-^EpCAM^-^CD45^+^CD11b^+^F4/80^+^Ly^-^6Chi), granulocytes (EpCAM^-^CD45^+^CD11b^+^Ly^-^6G^+^) and B lymphocytes (EpCAM^-^CD45^+^CD11b^-^CD19^+^) were identified as separate populations. (**b**) Flow cytometry analysis of individual leukocyte populations as a percent of total CD45^+^ cells in MMTV-PyMT/BC-PyMT and MMTV-Erbb2/BC-Erbb2 mammary tumors and in 20 PDX. The data shown are the mean percentages of viable cells ± SEM (standard error of the mean) for three mice per cohort. BC models were compared in Kruskal-Wallis tests. (**c**) Comparison of dissociated cell profiles showing the similarity of spontaneous and allografted MMTV-PyMT/BC-PyMT and MMTV-Erbb2/BC-Erbb2 tumors on the basis of anti-EpCAM, CD45, Ly-6C and F4/80 staining. (**d**) Specific stromal profiles of BC tumors. Clustering of stromal population percentage data from flow cytometry shown in (2b) above.

As previously reported [11, 18, 25], the tumor cell characteristics of the original patient tumors, such as cellularity, morphology, and architecture (tumor and stromal cell distribution and *in situ* tissue morphology), were highly conserved in the corresponding PDXs (**Fig 1A**) (**S7 Fig**). Masson’s trichrome staining showed low levels of fibrosis in PDXs (**Fig 2A**). Strong myofibroblast infiltration and organization were observed in these models (HBCx-3/13B). Vascular development differed between models (**Fig 1A**) (**S8–S10 Figs).**

### Histological characterization of paired GEMs and the corresponding tumors transplanted into immunodeficient mice

We compared stromal components between the spontaneous tumors (MMTV-PyMT and MMTV-ErbB2) and the corresponding allografted tumors in immunodeficient mice (BC-PyMT and BC-ErbB2). The spontaneous tumors and the corresponding allografts had very similar stromal features in terms of tissue morphology, fibrosis, myofibroblast infiltration, and vasculature (**Fig 1B)**.

### Each PDX is defined by a unique hematopoietic mouse-derived stroma

Hematopoietic stromal cells were analyzed by flow cytometry in a total of 21 PDXs, including triple-negative, HER2-positive and luminal B-cell tumor models (**S1 Table**). A gating strategy was developed to distinguish between different subtypes of hematopoietic cells (**Fig 2A**). By contrast to our immunohistochemistry results, but in accordance with other studies [33], flow-cytometry analysis of CD45^+^ cells showed that most of the stromal cells were of hematopoietic origin (**S11 Fig**).

Stromal heterogeneity, as measured by flow cytometry (FC), differed between models (**Fig 2B**). Seven of 21 tumors had a high percentage (at least 40%) of macrophage-like cells, 6 had 20–40% macrophage-like cells, and eight had fewer than 20% macrophage-like cells (**Fig 2B**). In some models, stromal infiltration predominantly involved granulocytes (HBCx-41) or B lymphocytes (HBCx-3 and -22). Stromal analysis revealed a specific fingerprint for each model, with two distinct clusters defined principally on the basis of macrophage infiltration (**Fig 2D**). Due to the small number of HER2 and luminal B-cell PDXs, no significant association between a particular BC subtype and a specific type of stroma was identified. Due to the fact that it was not possible to include in each cytometry analysis the PyMT model positive control, each PDX was at least studied in three independent experiments, with very restrictive error bars (**Fig 2B**).

### The hematopoietic cell composition of mouse-derived stroma is conserved between GEM tumors and their corresponding transplanted tumors in immunodeficient mice

Hematopoietic stromal cells were analyzed by flow cytometry in two GEMs (MMTV-PyMT and MMTV-ErBb2) and the corresponding transplanted tumors in immunodeficient *Nude* mice (BC-PyMT and BC-erBb2). The proportions of stromal hematopoietic cells in the spontaneous MMTV-PyMT and MMTV-ErbB2 tumors were similar to those in their allografted counterparts, with a high level of macrophage infiltration (**Fig 2C and 2D**). Stromal composition was strongly correlated between the original and allografted tumors (**Fig 2D)**.

### The macrophage-like populations in PDX and GEM models are defined by specific individual gene expression patterns

Macrophages can be polarized towards M1 or M2 populations in response to environmental signals. M2 macrophages play a key role in the subversion of adaptive immunity and in the inflammatory circuits promoting tumor growth and progression. MMTV-PyMT macrophages have been studied in detail and have recently been implicated in lung metastasis [34].

Five tumor models in which macrophage-like cells predominated (MMTV-PyMT, the corresponding allograft BC-PyMT and 3 PDXs (HBCx-5/24/34)) were chosen for further analysis (**Fig 2B**). Macrophage-like cells (three tumors per model) were purified by cell sorting and their expression profiles examined (**S1–S6 Figs**).

**Fig 3:**
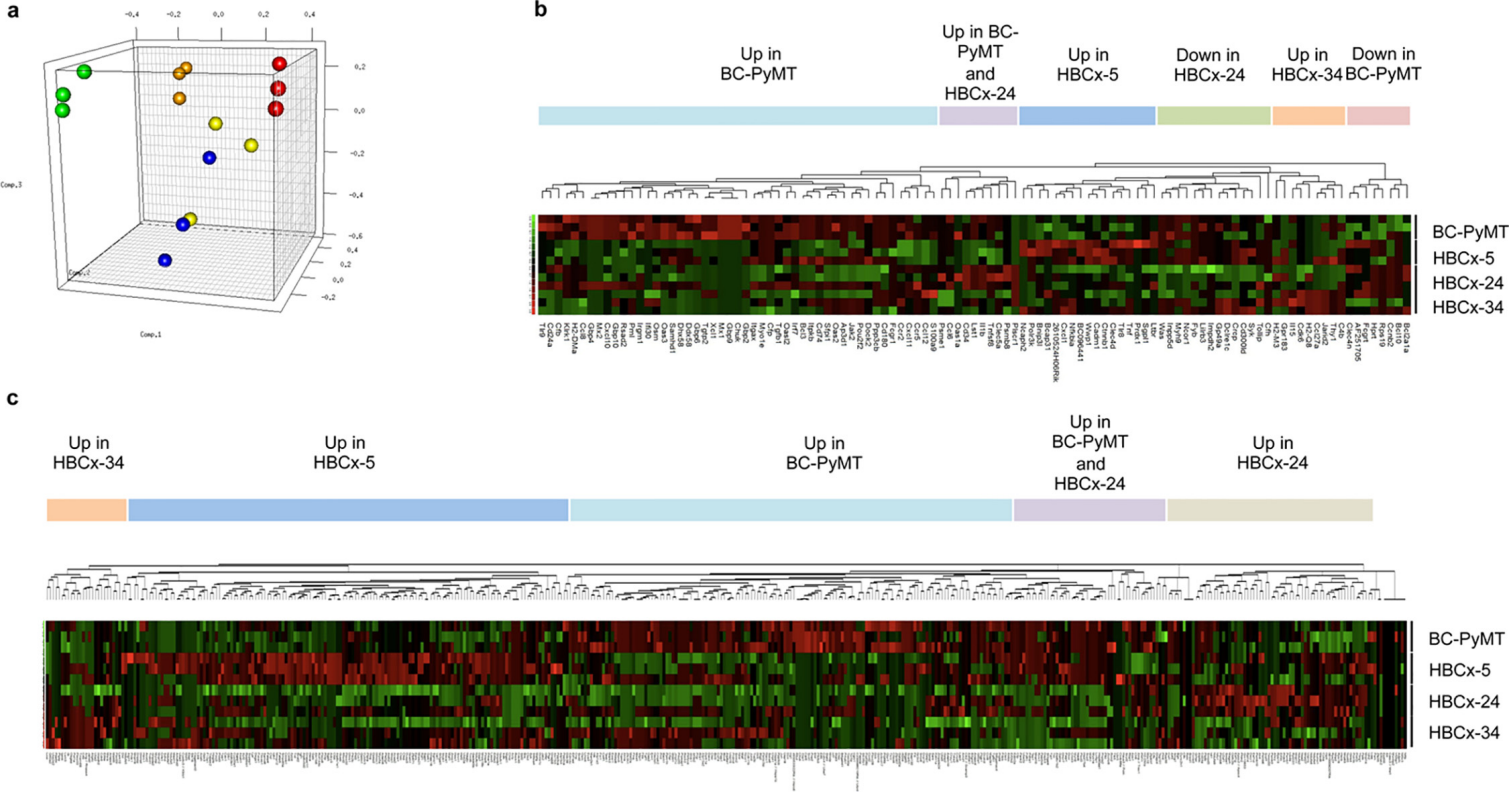
Transcriptome profiles of macrophage-like cells in BC tumors. (**a**) Results of principal component analysis (PCA) for the subset of 1238 genes up- or downregulated in at least one comparison between MMTV-PyMT and BC-PyMT, HBCx-5, -24 or -34. The 15 samples, triplicates of MMTV-PyMT (green), BC-PyMT (orange), HBCx-5 (red), HBCx-24 (yellow) and HBCx-34 (blue), were projected oton the first three principal components, which accounted for ~58% of the total variability. Hierarchical clustering of the genes from the (**b**) “Immune system process” or (**c**) “Metabolic process” pathways from Gene Ontology analysis identified as significantly up- or downregulated in at least one comparison between BC-PyMT and HBCx-5, -24 and -34.

Gene expression profiles of macrophage-like cells sorted from the spontaneous mouse MMTV-PyMT tumor were compared with those of the corresponding allograft (BC-PyMT) and the three human PDX models. The triplicates tested for each model clustered together on three-dimensional principal component analysis (PCA) (**Fig 3A**). In addition, the macrophage-like cells from the transplanted BC-PyMT tumor were closer to those of transplanted PDXs than to those of the original spontaneous MMTV-PyMT. This finding was confirmed by unsupervised clustering analysis (data not shown).

An analysis of gene expression in the spontaneous MMTV-PyMT tumor and the corresponding allograft, BC-PyMT, showed 486 genes to be differentially expressed between these two tumors (**S4 Table**). Using the mouse Gene Ontology (GO) pathway gene sets, we identified “Immune response” and “Immune system process” as the principal GO pathways enriched in the BC-PyMT allograft (**S5 Table**). Analyses of up- and downregulated genes revealed a strong interferon signature, with many interferon-stimulated genes, encoding cytokines/chemokines (*Ccl2*, *Cxcl11* and *Cxcl10*), regulatory proteins (*STAT*, *Slfn*), enzymes (*Oas*, *Mx*, *Igtp*), lymphocyte antigens, nucleotide binding proteins and major histocompatibility complex (MHC) molecules (**S6 Table**) expressed more strongly in the allograft than in the original tumor. This upregulation of the interferon pathway presumably reflects allograft rejection [35].

For the identification of genes specific to each model and not upregulated due to graft rejection, we compared transplanted mouse tumors directly with human tumors transplanted into mice. Macrophage-like cells from mouse BC-PyMT were compared with PDXs. Differential expression was observed for 414 (HBCx-24 and -34) and 540 genes (HBCx-5) (**S4 Table**). Both the “Immune system process” and “Immune response” GO pathways were found to display differential expression between PDXs and BC-PyMT (**S7 and S8 Tables**).

The clustering of “Immune system process”-related genes showed BC-PyMT macrophage-like cells to be defined by high levels of expression for 32 genes, including *Cxcl10* and other interferon-stimulated genes (*OAS*, *Mx*, *Gbp*, *Irf7*) (**Fig 3B**). The expression of interferon-related genes was also upregulated in PDX xenografts, but less strongly than in the allografts. Macrophage-like cells from each PDX model were then compared with the BC-PyMT allograft. In the HBCx-5 model, eight genes were specifically upregulated; these genes encoded Toll-like receptors (*Tlr8*), monocyte/macrophage-specific C-type lectins (CLEC) (*Clec4d*) and cell adhesion molecules (*Cadm1*). In HBCx-34, high levels of expression were observed for genes encoding several cytokines (*Il15*, *Ccl12* and *Ccl27a*) and chemokine receptors (*Ccr2* and *Ccr5*). HBCx-24 displayed no unique clusters but several genes were upregulated in this PDX and BC-PyMT (*Ccl6*, *Ccl8*, *Il1b*, and others) or HBCx-5 (*Fcgrt*, *Clec4n*).

GO analyses identified several non-immune related pathways common to the PDXs and BC-PyMT. For the “Metabolic process” pathway, 207, 159 and 151 genes in HBCx-5, -24 and -34, respectively, were expressed to levels different from those in BC-PyMT. (**S8 Table**). In total, 450 genes were differentially expressed relative to BC-PyMT in at least one of the PDXs relative to BC-PyMT. These 450 genes formed five distinct clusters: four specific to the various models and one cluster common to BC-PyMT and HBCx-24 (**Fig 3C**). The largest differences were observed in HBCx-5, in which many metabolism-related genes, including genes encoding cytochromes (*Cyba*, *Cybr5r3*, *Cyp20&1* and *Cyp4v3*), aldehyde dehydrogenases (*Aldh2*, *Aldh3b1*), cathepsins (*cathepsin B*, *D*, *K and L*) and other enzymes (*Galk1*, *Glb1*, *Galc*, *Gnpda1*), were found to be differentially expressed.

Thus, the macrophage-like cells from the BC-PyMT allograft and HBCx-5, -24, and -34 xenograft models have different specific gene expression profiles.

### Macrophage-like cells from PDXs and GEMs display heterogeneous expression of M1 and M2 markers

The key role of tumor-associated macrophages (TAMs) in tumors is dependent on their M1 or M2 activation. We determined the macrophage-like cell activation phenotype, by sorting macrophage-like cells from MMTV-PyMT/BC-PyMT and PDXs and evaluating gene expression and protein levels for well-defined M1 and M2 markers [36, 37].

**Fig 4:**
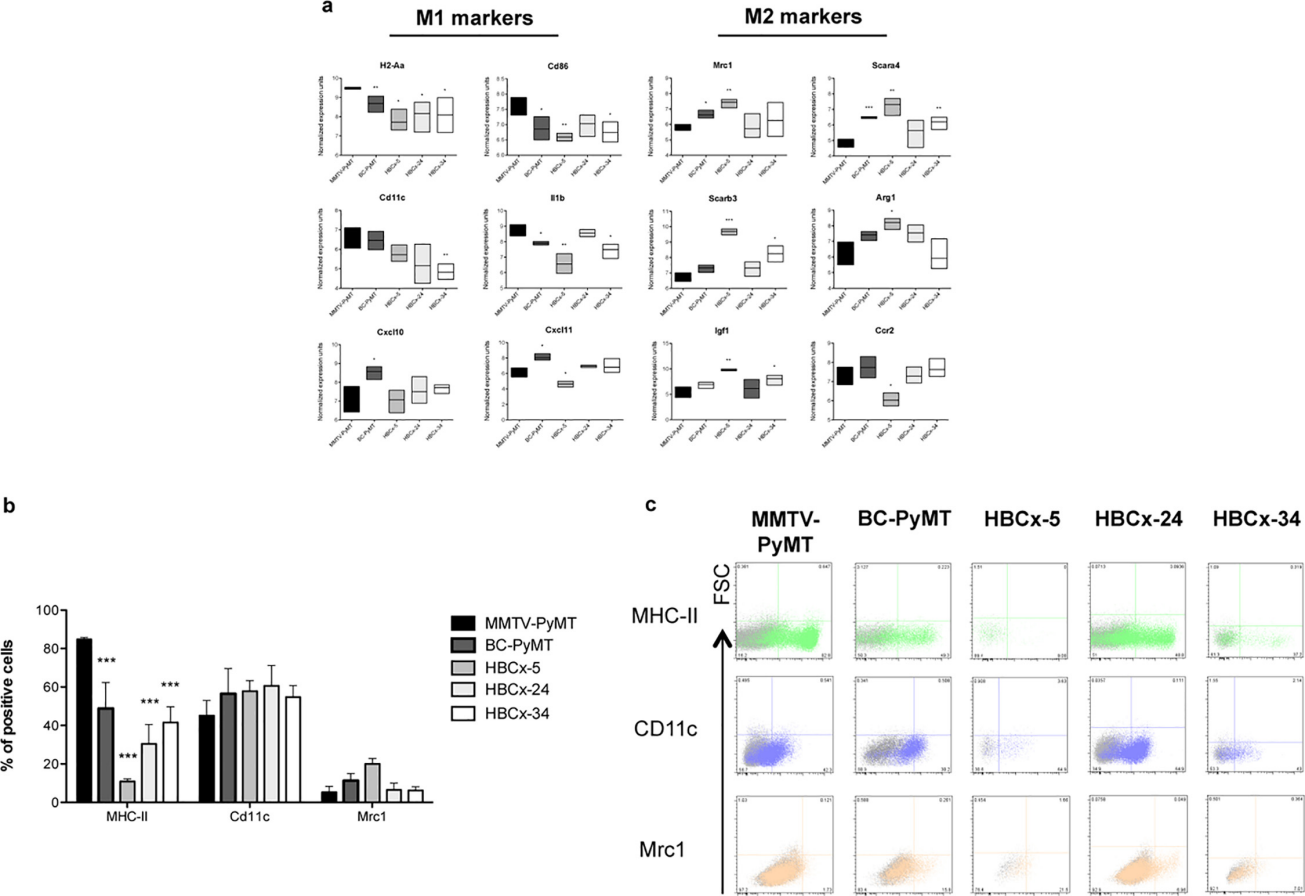
M1/M2 macrophage-like cell phenotype in BC tumors. (**a**) The expression of genes associated with the M1 (MHC-II, CD86, Cd11c, Il1b, Cxcl10 and Cxcl11) or M2 (Mrc1, Scara4, Scarb3, Arg1, Igf1 and Ccr2) phenotype was assessed by the dissociation of five tumors (MMTV-PyMT, BC-PyMT, HBCx-5, -24 and -34), the sorting of macrophage-like cells, and microarray analysis. Levels of gene expression in BC models were compared in unpaired Student’s *t*-tests (**b**) Protein levels for M1 (MHC-II and Cd11c) and M2 (Mrc1) markers on macrophage-like cells from the five tumors MMTV-PyMT, BC-PyMT, HBCx -5, -24 and x-34), as measured by flow cytometry. Three tumors were analyzed per model. For each model, Mann-Whitney tests were performed to compare the results obtained with those for the MMTV-PyMT tumor (* *p*< 0.05, **p <0.01, ***p <0.001). (**c**) Examples of flow cytometry findings for the levels of M1 and M2 marker proteins (the corresponding isotype is shown in gray).

M1 marker genes were more strongly expressed, and M2 markers less strongly expressed in the mouse spontaneous MMTV-PyMT tumor than in all the other models (*p*<0.05). The mouse BC-PyMT allograft overexpressed the M1 marker genes *Cxcl10* and *Cxl11* (**Fig 4A**). In the HBCx-5 model, the M2 marker genes, *Mrc1* (3.15-fold; *p*<0.01), *Scara4* (5.46-fold; *p*<0.01), *Scarb3* (7.53-fold; *p*<0.001) and *Arginase 1* (3.88-fold; *p*<0,05) were particularly strongly expressed, whereas *Ccr2* was underexpressed.

Protein levels for three M1 and M2 membrane proteins—MHC-II, CD11C and MRC1 (**Fig 4B and 4C**)—were assessed by flow cytometry. Consistent with gene expression levels, MHC-II (M1) protein levels were significantly higher in MMTV-PyMT than in the other models (*p*<0.001). MHC-II protein levels were higher in BC-PyMT than in the PDXs. CD11C (M1) protein levels were similar in all models. MRC1 (M2) levels were higher in BC-PyMT and HBCx-5 than in MMTV-PyMT.

## Discussion

The aim of this study was to characterize the tumor-associated stroma in both human and mouse breast cancer models (i.e. GEMs and PDXs), and to determine the impact of subcutaneous transplantation on stromal components in immunodeficient mice.

Consistent with previous data, the unique architecture and cellular morphology, and the genomic and gene expression profiles of the original tumor cells were conserved in PDXs [19, 25]. We also observed infiltration with myofibroblasts in particular, and heterogeneous levels of vascularization in human xenografts, reflecting effective partial crosstalk between human tumor cells and mouse stromal cells resulting in the recruitment of myofibroblasts and endothelial cells [38].

An analysis of the hematopoietic compartment in tumors showed that each PDX model had its own unique stromal cell profile. However, macrophages and granulocytes predominated, reflecting local inflammation. This feature had already been reported in some transgenic mouse models such, as MMTV-PyMT, in which macrophages predominate, but our data highlighted the variability of the hematopoietic cell component in PDXs, as in human tumors[34, 39]. The reproducibility of our results for each tumor suggests that this variability between models corresponds to a relevant specific stromal fingerprint [27]. It has to be mentioned that, despite a high rate of macrophages detected by cytometry analyses in various PDXs, we have also detected a relatively low rate of infiltrating macrophages in corresponding tumor model’s tissue sections, suggesting a limitation of IHC method in the study of macrophage polarization. Finally, the number of hematopoietic stromal cells was similar to that observed in spontaneous or subcutaneously allotransplanted GEM. Transplantation into immunodeficient mice therefore had no effect on the stromal cell profile of the mouse model, as previously reported [8].

An analysis of the gene expression profiles of sorted macrophage-like cells revealed a basic interferon signature in all the models studied, potentially related to the allograft process [40]. However, we could not formally exclude the possibility that interferon-stimulated genes were differentially expressed due to a lack of functional T cells. Further studies involving the injection of PyMT cells into immune competent mice would be required to resolve this issue. However, each of the preclinical models studied—MMTV- and BC-PyMT and PDXs—had its own individual gene expression profile. For instance, macrophage-like cells sorted from MMTV-PyMT displayed strong M1 differentiation, as previously described [41], whereas PDX models were more heterogeneous, with an upregulation of typical M1/M2-specific genes, such as those encoding MHC-II, scavenger receptors, or arginases [36]. Pathways involved in tumor growth, invasion and metastasis were differentially expressed in following models: (1) HBCx-34 overexpressed *Ccl12* inflammation-associated chemokines and *Il15*, encoding IL-15, which has been shown to inhibit tumor growth in murine models via natural killer (NK) cells [42]; (2) the pro-tumorigenic interleukin-1 beta was overproduced in both HBCx-24 and BC-PyMT, indicating a phenotype common to murine and human tumors [43]; (3) genes differentially expressed between PDXs and PyMT, such as *Mmp12*, which has been implicated in tumor progression, metastasis and angiogenesis in a lung carcinoma model [44, 45]; (4) in HBCx-5, specific spots were obtained for metabolism-related genes and levels of cysteine cathepsins were high, and high levels of two cathepsins, cathepsins B and L, have been correlated with poor survival in cancer patients [46]. All these observations indicate that stromal characterization in individuals may identify tumor-specific pathways leading to tumor progression and dissemination that could be targeted for treatment.

### Conclusions

In conclusion, our results show that each PDX and GEM tumor can be defined by its individual tumor-associated stromal matrix. Tumor cells can thus generate their own specific stromal composition, despite the absence of T cells, and maintain a complex functional network of communications. Models such as those described here may therefore be considered relevant tools for preclinical and pharmacological assessment to investigate tumor and stromal interactions, but further studies are required to determine the precise role of these stromal cells in tumor development.

## Supporting Information

S1 Fig Fluorescence-activated cell sorting of F4/80^+^ macrophage-like cells from MMTV-PyMT tumors. (PDF)

S2 Fig Fluorescence-activated cell sorting of F4/80^+^ macrophage-like cells from BC-PyMT tumors. (PDF)

S3 Fig Fluorescence-activated cell sorting of F4/80^+^ macrophage-like cells from HBCx-5 tumors. (PDF)

S4 Fig Fluorescenc- activated cell sorting of F4/80^+^ macrophage-like cells from HBCx-24 tumors. (PDF)

S5 Fig Fluorescence-activated cell sorting of F4/80^+^ macrophage-like cells from HBCx-34 tumors. (PDF)

S6 Fig MGG-stained cytospins of purified macrophage-like cells. (PDF)

S7 Fig Histology of human breast cancer tumors and corresponding xenografts. (PDF)

S8 Fig Fibrosis in human breast cancer tumors and corresponding xenografts. (PDF)

S9 Fig Myofibroblasts in human breast cancer tumors and corresponding xenografts. (PDF)

S10 Fig Endothelial cells in human breast cancer tumors and corresponding xenografts. (PDF)

S11 Fig Flow cytometry analysis of cells harvested after tumor dissociation. (PDF)

S1 Table Biological characteristics of HBC xenografts. (PDF)

S2 Table Primary antibodies used for immunohistochemistry. (PDF)

S3 Table Primary antibodies used in FC analysis. (PDF)

S4 Table Genes differentially expressed in macrophage-like cells purified from MMTV-PyMT, BC-PyMT, HBCx-5, HBCx-24, and HBCx-34 tumor grafts. (PDF)

S5 Table Gene Ontology analysis and comparison of macrophage-like cells purified from MMTV-PyMT and BC-PyMT. (PDF)

S6 Table Interferon-stimulated genes (ISGs) up- or downregulated in macrophage-like cells isolated from BC-PyMT relative to MMTV-PyMT. (PDF)

S7 Table Differentially regulated GO pathways in macrophage-like cells purified from MMTV-PyMT, BC-PyMT, HBCx-5, HBCx-24, and HBCx-34 tumor grafts. (PDF)

S8 Table Gene Ontology analysis of genes differentially expressed in BC-PyMT relative to HBCx-5, HBCx-24 and HBCx-34 TAMs. (PDF)
