# Supplementary material for: Characterization of Breast Cancer Preclinical Models Reveals a Specific Pattern of Macrophage Polarization
Source: PLoS One. 2016 Jul 7;11(7):e0157670. doi: 10.1371/journal.pone.0157670 (PMC4936680; doi:10.1371/journal.pone.0157670)
Supplement: S1 Fig — (PDF) [file pone.0157670.s001.pdf]

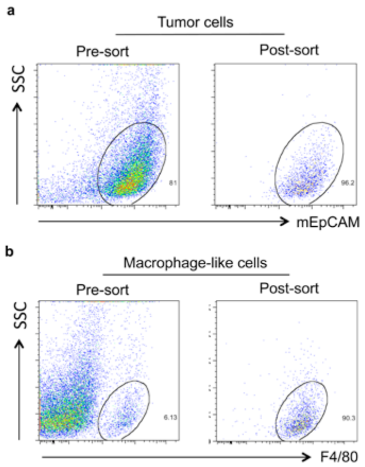

Supplementary Figure 1. Fluorescence activated cell sorting of F4/80+ macrophage-like cells. Purification of F4/80+ macrophage-like cells from MMTV-PyMT tumors by FACS sorting of CD45+F4/80+ positive cells. Representative analyses of pre- and post-sort macrophage-like cell populations.
