# Supplementary material for: Characterization of Breast Cancer Preclinical Models Reveals a Specific Pattern of Macrophage Polarization
Source: PLoS One. 2016 Jul 7;11(7):e0157670. doi: 10.1371/journal.pone.0157670 (PMC4936680; doi:10.1371/journal.pone.0157670)
Supplement: S6 Fig — (PDF) [file pone.0157670.s006.pdf]

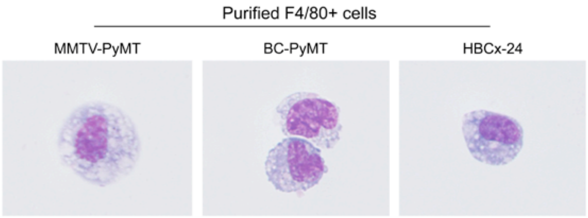

Supplementary Figure 6. MGG-stained cytospins of purified macrophage-like cells. CD45+F4/80+ macrophage-like cells purified from (**a**) MMTV-PyMT, (**b**) BC-PyMT and (**c**) HBCx-24 BC models were stained with MGG. Macrophage-like cells displayed vacuoles identifying them as macrophages.
