# Supplementary material for: Characterization of Breast Cancer Preclinical Models Reveals a Specific Pattern of Macrophage Polarization
Source: PLoS One. 2016 Jul 7;11(7):e0157670. doi: 10.1371/journal.pone.0157670 (PMC4936680; doi:10.1371/journal.pone.0157670)
Supplement: S10 Fig — (PDF) [file pone.0157670.s010.pdf]

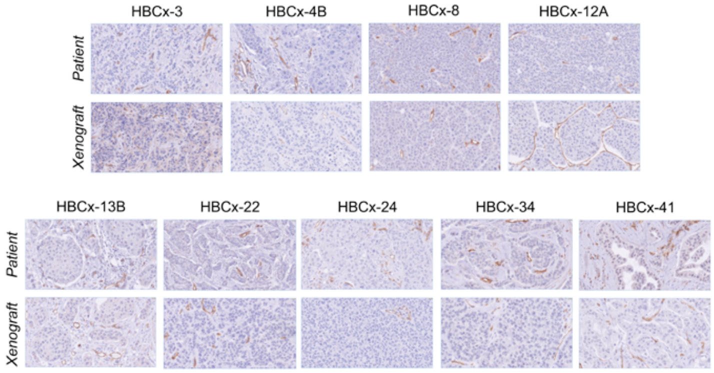

Supplementary Figure 10. Endothelial cells in human breast cancer tumors and corresponding xenografts. The original human tumors (H) and their corresponding xenografts (X) for 9 models were stained with CD31 (Original magnification 400x). Endothelial cells were assessed as the proportion of CD31 positive areas in the fields examined.
