# Supplementary material for: Characterization of Breast Cancer Preclinical Models Reveals a Specific Pattern of Macrophage Polarization
Source: PLoS One. 2016 Jul 7;11(7):e0157670. doi: 10.1371/journal.pone.0157670 (PMC4936680; doi:10.1371/journal.pone.0157670)
Supplement: S11 Fig — (PDF) [file pone.0157670.s011.pdf]

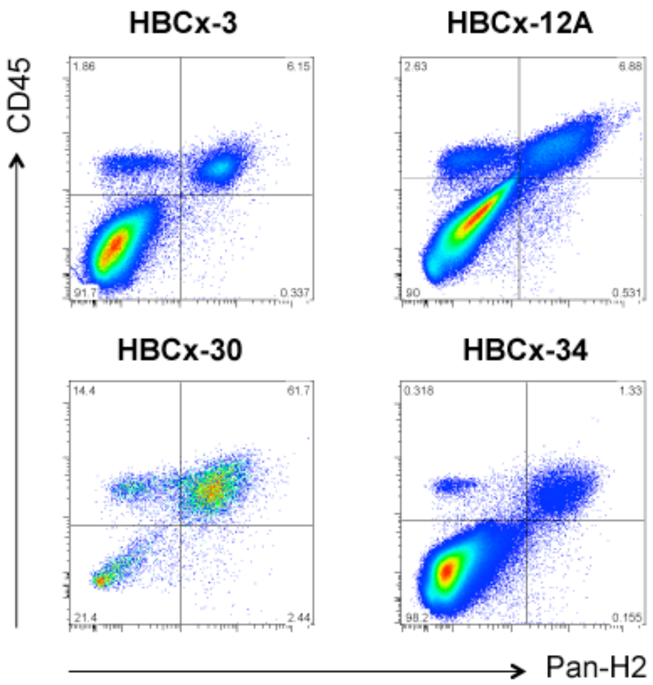

Supplementary Figure 11. Flow cytometric analysis of cells harvested after tumor dissociation. Representative flow cytometric analysis of viable dissociated cells from 4 models of BC human xenografts (i.e HBCx-3, -12A, -30 and -34) stained with murine specific CD45 and MHC-I molecules (Pan H-2) markers.
