# Supplementary material for: Characterization of Breast Cancer Preclinical Models Reveals a Specific Pattern of Macrophage Polarization
Source: PLoS One. 2016 Jul 7;11(7):e0157670. doi: 10.1371/journal.pone.0157670 (PMC4936680; doi:10.1371/journal.pone.0157670)
Supplement: S1 Table — (PDF) [file pone.0157670.s012.pdf]

Model	Histology	Primary/Metastasis	IHC xenograft	ERa	PR	HER2	Ki67	Stroma analysis (Microarray)	Stroma analysis (IHC)	Stroma analysis (FC)	Macrophage analysis (Microarray)
HBCx-1	IDC	Primary	Triple negative	78	0	4416	7724	X			
HBCx-10	IDC	Primary	Triple negative	190	0	1622	1640	X		X	
HBCx-11	IDC	Primary	Triple negative	0	0	1347	3509	X			
HBCx-12A	IDC	Primary	Triple negative	67	1	5548	7040	X	X	X	
HBCx-12B	IDC	Metastasis	Triple negative	UK	UK	UK	UK	X		X	
HBCx-13B	IDC	Metastasis	HER2	UK	UK	UK	UK	X	X	X	
HBCx-14	IDC	Primary	Triple negative	35	0	301	4158			X	
HBCx-15	Undifferentiated	Primary	Triple negative	15	0	1056	5484	X		X	
HBCx-16	IDC	Primary	Triple negative	UK	10	7119	UK			X	
HBCx-19	ILC	Metastasis	Luminal	UK	UK	UK	UK	X			
HBCx-21	Mucinous	Primary	Luminal	3953	370	1095	2151			X	
HBCx-22	IDC	Primary	Luminal	4619	798	10846	8303		X	X	
HBCx-23	IDC	Primary	Triple negative	17	4	1010	2498			X	
HBCx-24	IDC	Primary	Triple negative	13	0	1233	7810		X	X	X
HBCx-3	IDC	Primary	Luminal	1381	16	563	5592	X	X	X	
HBCx-30	IDC	Primary	Triple negative	2	0	3068	9353			X	
HBCx-34	IDC	Primary	Luminal	1789	147	5041	6179		X	X	X
HBCx-39	IDC	Primary	Triple negative	UK	UK	UK	UK			X	
HBCx-40	IDC	Primary	Triple negative	UK	UK	UK	UK			X	
HBCx-41	Micropapillary	Primary	HER2	UK	UK	UK	UK		X	X	
HBCx-4B	IDC	Metastasis	Triple negative	325	0	460	8046		X	X	
HBCx-5	Colloid	Metastasis	HER2	986	0	78607	1532			X	X
HBCx-6	IDC	Primary	Triple negative	34	0	1480	4507	X			
HBCx-7	ILC	Metastasis	Triple negative	0	0	370	1086	X			
HBCx-8	IDC	Primary	Triple negative	1	0	676	1152		X	X	
HBCx-9	IDC	Primary	Triple negative	2	0	1762	1148			X	

Supplementary Table 1. Biological characteristics of HBC xenografts.

Quantitative RT-PCR was performed for expression analysis.
