# Supplementary material for: Characterization of Breast Cancer Preclinical Models Reveals a Specific Pattern of Macrophage Polarization
Source: PLoS One. 2016 Jul 7;11(7):e0157670. doi: 10.1371/journal.pone.0157670 (PMC4936680; doi:10.1371/journal.pone.0157670)
Supplement: S2 Table — (PDF) [file pone.0157670.s013.pdf]

Supplementary Table 2: Primary antibodies used for immunohistochemistry

Antigen detected	Specificity	Clone	Source
a-SMA	Human/Mouse	1A4	Dako
CD31	Human/Mouse	Polyclonal	Neomarker
