# Supplementary material for: Characterization of Breast Cancer Preclinical Models Reveals a Specific Pattern of Macrophage Polarization
Source: PLoS One. 2016 Jul 7;11(7):e0157670. doi: 10.1371/journal.pone.0157670 (PMC4936680; doi:10.1371/journal.pone.0157670)
Supplement: S3 Table — (PDF) [file pone.0157670.s014.pdf]

Supplementary Table 3: Primary antibodies used in FC analysis

Antigen detected	Fluorochrome	Clone	Source
CD11b	AF700	M1/70	BDBiosciences
CD11c	PE-Cy7	N418	Ebioscience
CD19	APC-H7	1D3	BDBiosciences
CD206	AF488	MR5D3	Biolegend
CD45	PE-TR	30-F11	Invitrogen
hEpCAM	PerCP-Cy5.5	9C4	Biolegend
mEpCAM	PerCP-eFluor710	G8.8	Ebioscience
F4/80	APC	BM8	Ebioscience
Ly-6C	APC-Cy7	AL-21	BDBiosciences
Ly-6G	FITC	1A8	BDBiosciences
MHC-II	APC-H7	M5/114.15.2	Biolegend
Pan-H2	PE	M1/42	Biolegend
