# Supplementary material for: Characterization of Breast Cancer Preclinical Models Reveals a Specific Pattern of Macrophage Polarization
Source: PLoS One. 2016 Jul 7;11(7):e0157670. doi: 10.1371/journal.pone.0157670 (PMC4936680; doi:10.1371/journal.pone.0157670)
Supplement: S4 Table — (PDF) [file pone.0157670.s015.pdf]

Supplementary Table 4: Differentially expressed genes of macrophages-like cells purified from MMTV-PyMT, BC-PyMT, HBCx-5, HBCx-24, and HBCx-34 tumor grafts

	BC-PyMT			HBCx-5			HBCx-24			HBCx-34		
	All	Up	Down	All	Up	Down	All	Up	Down	All	Up	Down
**MMTV-PyMT**	486	250	236	492	292	200	325	86	239	409	127	282
**BC-PyMT**				540	330	210	414	140	274	414	123	291
**HBCx-5**							468	109	359	393	85	308
**HBCx-24**										236	115	121
