# Supplementary material for: Characterization of Breast Cancer Preclinical Models Reveals a Specific Pattern of Macrophage Polarization
Source: PLoS One. 2016 Jul 7;11(7):e0157670. doi: 10.1371/journal.pone.0157670 (PMC4936680; doi:10.1371/journal.pone.0157670)
Supplement: S5 Table — (PDF) [file pone.0157670.s016.pdf]

Supplementary Table 5: Gene Ontology analysis of macrophage-like cells purified from MMTV-PyMT *vs* BC-PyMT.

Term Type	GO ID with Link	Go Term	Nb Regulated Genes (Up / Down)	P-Value
biological_process	GO:0006955	immune response	37 (26/11)	3,47E-13
biological_process	GO:0002376	immune system process	47 (30/17)	9,22E-12
cellular_component	GO:0005737	cytoplasm	177 (83/94)	2,68E-09
cellular_component	GO:0044424	intracellular part	221 (107/114)	3,73E-06
cellular_component	GO:0005622	intracellular	225 (109/116)	5,54E-06
biological_process	GO:0010573	vascular endothelial growth factor production	3 (1/2)	1,14E-05
biological_process	GO:0010574	regulation of vascular endothelial growth factor production	3 (1/2)	1,14E-05
biological_process	GO:0010575	positive regulation vascular endothelial growth factor production	3 (1/2)	1,14E-05
cellular_component	GO:0043231	intracellular membrane-bounded organelle	174 (82/92)	3,07E-05
cellular_component	GO:0043227	membrane-bounded organelle	174 (82/92)	3,21E-05
cellular_component	GO:0044444	cytoplasmic part	105 (35/70)	4,37E-05
molecular_function	GO:0019239	deaminase activity	5 (4/1)	4,55E-05
biological_process	GO:0006952	defense response	22 (16/6)	4,61E-05
molecular_function	GO:0001730	2'-5'-oligoadenylate synthetase activity	3 (3/0)	1,00E-04
biological_process	GO:0045087	innate immune response	10 (9/1)	3,00E-04
molecular_function	GO:0003735	structural constituent of ribosome	7 (0/7)	3,00E-04
molecular_function	GO:0004000	adenosine deaminase activity	3 (3/0)	3,00E-04
biological_process	GO:0050896	response to stimulus	65 (42/23)	4,00E-04
biological_process	GO:0002335	mature B cell differentiation	3 (2/1)	4,00E-04
cellular_component	GO:0005840	ribosome	9 (0/9)	4,00E-04
biological_process	GO:0001767	establishment of lymphocyte polarity	2 (2/0)	5,00E-04
biological_process	GO:0001768	establishment of T cell polarity	2 (2/0)	5,00E-04
biological_process	GO:0046060	dATP metabolic process	2 (1/1)	5,00E-04
molecular_function	GO:0003692	left-handed Z-DNA binding	2 (2/0)	5,00E-04
cellular_component	GO:0000313	organellar ribosome	5 (0/5)	5,00E-04
cellular_component	GO:0005761	mitochondrial ribosome	5 (0/5)	5,00E-04
molecular_function	GO:0003824	catalytic activity	118 (66/52)	6,00E-04
biological_process	GO:0002699	positive regulation of immune effector process	5 (5/0)	7,00E-04
biological_process	GO:0051707	response to other organism	12 (9/3)	9,00E-04
biological_process	GO:0006412	translation	14 (1/13)	9,00E-04
molecular_function	GO:0016814	hydrolase activity, acting on carbon-nitrogen (but not peptide) bonds, in cyclic amidines	4 (4/0)	1,00E-03
cellular_component	GO:0043229	intracellular organelle	183 (88/95)	1,00E-03
cellular_component	GO:0043226	organelle	183 (88/95)	1,00E-03
molecular_function	GO:0019205	nucleobase-containing compound kinase activity	4 (2/2)	1,20E-03
molecular_function	GO:0003953	NAD+ nucleosidase activity	2 (1/1)	1,40E-03
molecular_function	GO:0003726	double-stranded RNA adenosine deaminase activity	2 (2/0)	1,40E-03
cellular_component	GO:0005759	mitochondrial matrix	5 (0/5)	2,20E-03
cellular_component	GO:0005739	mitochondrion	40 (9/31)	2,70E-03

molecular_function	GO:0016833	oxo-acid-lyase activity	2 (0/2)	2,80E-03
molecular_function	GO:0042605	peptide antigen binding	2 (0/2)	2,80E-03
molecular_function	GO:0003950	NAD+ ADP-ribosyltransferase activity	3 (3/0)	3,20E-03
molecular_function	GO:0003743	translation initiation factor activity	5 (1/4)	3,30E-03
cellular_component	GO:0009986	cell surface	12 (7/5)	3,70E-03
molecular_function	GO:0019206	nucleoside kinase activity	2 (1/1)	4,60E-03
cellular_component	GO:0000315	organellar large ribosomal subunit	3 (0/3)	4,60E-03
cellular_component	GO:0005762	mitochondrial large ribosomal subunit	3 (0/3)	4,60E-03
cellular_component	GO:0005783	endoplasmic reticulum	28 (10/18)	4,60E-03
molecular_function	GO:0008137	NADH dehydrogenase (ubiquinone) activity	3 (0/3)	4,80E-03
molecular_function	GO:0016799	hydrolase activity, hydrolyzing N-glycosyl compounds	3 (1/2)	4,80E-03
molecular_function	GO:0050136	NADH dehydrogenase (quinone) activity	3 (0/3)	4,80E-03
molecular_function	GO:0003954	NADH dehydrogenase activity	3 (0/3)	4,80E-03
