# Supplementary material for: Characterization of Breast Cancer Preclinical Models Reveals a Specific Pattern of Macrophage Polarization
Source: PLoS One. 2016 Jul 7;11(7):e0157670. doi: 10.1371/journal.pone.0157670 (PMC4936680; doi:10.1371/journal.pone.0157670)
Supplement: S6 Table — (PDF) [file pone.0157670.s017.pdf]

Supplementary Table 6: Interferon-stimulated genes (ISGs) up- or down-regulated in macrophage-like cells isolated from BC-PyMT *vs* MMTV-PyMT.

EnsEMBL ID	Gene Symbol	Gene Name	Regulation	Fold-Change	P-Value
Cytokines/Chemokines					
ENSMUSG00000027399	Il1a	interleukin 1 alpha	down	3,83	6,28E-03
ENSMUSG00000027398	Il1b	interleukin 1 beta	down	1,72	0,0248
ENSMUSG00000035385	Ccl2	chemokine (C-C motif) ligand 2	up	2,03	1,86E-02
ENSMUSG00000060183	Cxcl11	chemokine (C-X-C motif) ligand 11	up	4,53	0,0116
ENSMUSG00000034855	Cxcl10	chemokine (C-X-C motif) ligand 10	up	2,65	0,0349
Regulatory Proteins					
ENSMUSG00000028037	Ifi44	interferon-induced protein 44	up	10,57	0,000178
ENSMUSG00000026535 // ENSMUSG00000054203	Ifi202b // Ifi205	interferon activated gene 202B // interferon activated gene 205	up	4,54	0,000177
ENSMUSG00000026104	Stat1	signal transducer and activator of transcription 1	up	2,46	1,79E-02
ENSMUSG00000040033	Stat2	signal transducer and activator of transcription 2	up	3,96	0,0041
ENSMUSG00000000204	Slfn4	schlafen 4	up	6,61	0,0021
ENSMUSG00000054404	Slfn5	schlafen 5	up	2,38	1,73E-02
ENSMUSG00000026946	Nmi	N-myc (and STAT) interactor	up	2,72	6,06E-03
ENSMUSG00000025498	Irf7	interferon regulatory factor 7	up	4,17	0,00774
ENSMUSG00000074896	Ifit3	interferon-induced protein with tetratricopeptide repeats 3	up	4,13	0,000475
ENSMUSG00000054072	Iigp1	interferon inducible GTPase 1	up	3,56	0,00614
ENSMUSG00000026896	Ifih1	interferon induced with helicase C domain 1	up	3,39	0,00728
ENSMUSG00000073489	Ifi204	interferon activated gene 204	up	3,38	0,00024
ENSMUSG00000034459	Ifit1	interferon-induced protein with tetratricopeptide repeats 1	up	3,25	0,0082
ENSMUSG00000040328 // ENSMUSG00000078920	Ifi47 // Olfr56	interferon gamma inducible protein 47 // olfactory receptor 56	up	2,88	0,0333
ENSMUSG00000045932	Ifit2	interferon-induced protein with tetratricopeptide repeats 2	up	2,87	0,0121
ENSMUSG00000025492	Ifitm3	interferon induced transmembrane protein 3	up	2,81	0,0143
ENSMUSG00000079017	Ifi27l2a	interferon, alpha-inducible protein 27 like 2A	up	2,54	0,0118
ENSMUSG00000064215	Ifi27l1	interferon, alpha-inducible protein 27 like 1	down	1,72	4,69E-02
ENSMUSG00000001627	Ifrd1	interferon-related developmental regulator 1	up	1,51	0,0486
Enzyme					
ENSMUSG00000052776	Oas1a	2'-5' oligoadenylate synthetase 1A	up	1,74	0,0496
ENSMUSG00000041827	Oasl1	2'-5' oligoadenylate synthetase-like 1	up	3,98	0,0185
ENSMUSG00000029561	Oasl2	2'-5' oligoadenylate synthetase-like 2	up	3,87	0,0153
ENSMUSG00000032690	Oas2	2'-5' oligoadenylate synthetase 2	up	3,07	0,00278
ENSMUSG00000032661	Oas3	2'-5' oligoadenylate synthetase 3	up	2,99	0,00492
ENSMUSG00000023341	Mx2	myxovirus (influenza virus) resistance 2	up	2,47	0,00908

ENSMUSG00000000386	Mx1	myxovirus (influenza virus) resistance 1	up	2,3	0,0242
ENSMUSG00000078921	Tgtp2	T-cell specific GTPase 2	up	4,03	1,58E-02
ENSMUSG00000048852 // ENSMUSG00000078922	Gm12185 // Tgtp1	predicted gene 12185 // T-cell specific GTPase 1	up	3,97	0,00382
ENSMUSG00000046879	Irgm1	immunity-related GTPase family M member 1	up	2,68	9,62E-03
ENSMUSG00000078853	Igtp	interferon gamma induced GTPase	up	2,46	0,0359
ENSMUSG00000069874	Irgm2	immunity-related GTPase family M member 2	up	1,68	0,0487
Lymphocyte antigens					
ENSMUSG00000075602	Ly6a	lymphocyte antigen 6 complex, locus A	up	8,98	0,000364
ENSMUSG00000079018	Ly6c1	lymphocyte antigen 6 complex, locus C1	up	6,26	0,00228
ENSMUSG00000022587	Ly6e	lymphocyte antigen 6 complex, locus E	up	2,64	0,0306
ENSMUSG00000021423	Ly86	lymphocyte antigen 86	down	1,7	0,032
Receptors					
ENSMUSG00000015947	Fcgr1	Fc receptor, IgG, high affinity I	up	1,97	0,0177
ENSMUSG00000059089	Fcgr4	Fc receptor, IgG, low affinity IV	up	1,79	0,0105
Cell adhesion proteins					
ENSMUSG00000027962	Vcam1	vascular cell adhesion molecule 1	down	3,23	0,0147
Nucleotide binging protein					
ENSMUSG00000079363	Gbp4	guanylate binding protein 4	up	3,53	0,017
ENSMUSG00000079362	Gbp6	guanylate binding protein 6	up	3,12	0,0103
ENSMUSG00000029298	Gbp9	guanylate-binding protein 9	up	2,51	0,00866
ENSMUSG00000054588	Gbp10	guanylate-binding protein 10	up	1,72	0,0339
MHC components					
ENSMUSG00000056116	H2-T22 // H2-T9	histocompatibility 2, T region locus 22 // histocompatibility 2, T region locus 9	up	3,25	0,047
ENSMUSG00000055413	H2-Q8	histocompatibility 2, Q region locus 8	up	2,52	0,00602
ENSMUSG00000035929	H2-Q4	histocompatibility 2, Q region locus 4	up	2,09	0,00178
ENSMUSG00000079491	H2-T10	histocompatibility 2, T region locus 10	up	1,91	0,0256
ENSMUSG00000073421	H2-Ab1	histocompatibility 2, class II antigen A, beta 1	down	3,08	0,00554
ENSMUSG00000079547	H2-DMb1	histocompatibility 2, class II, locus Mb1	down	2,74	0,00258
ENSMUSG00000036594	H2-Aa	histocompatibility 2, class II antigen A, alpha	down	2,27	0,00524
ENSMUSG00000060802	B2m	beta-2 microglobulin	up	1,56	0,0384
ENSMUSG00000022901	Cd86	CD86 antigen	down	1,73	0,0465
