# Supplementary material for: Characterization of Breast Cancer Preclinical Models Reveals a Specific Pattern of Macrophage Polarization
Source: PLoS One. 2016 Jul 7;11(7):e0157670. doi: 10.1371/journal.pone.0157670 (PMC4936680; doi:10.1371/journal.pone.0157670)
Supplement: S7 Table — (PDF) [file pone.0157670.s018.pdf]

Supplementary Table 7: Differentially regulated GO pathways in macrophage-like cells purified from MMTV-PyMT, BC-PyMT, HBCx-5, HBCx-24, and HBCx-34 tumor grafts

	BC-PyMT	HBCx-5	HBCx-24	HBCx-34
**MMTV-PyMT**	51	301	109	182
**BC-PyMT**		371	161	227
**HBCx-5**			252	187
**HBCx-24**				109
