# Supplementary material for: Characterization of Breast Cancer Preclinical Models Reveals a Specific Pattern of Macrophage Polarization
Source: PLoS One. 2016 Jul 7;11(7):e0157670. doi: 10.1371/journal.pone.0157670 (PMC4936680; doi:10.1371/journal.pone.0157670)
Supplement: S8 Table — (PDF) [file pone.0157670.s019.pdf]

Supplementary Table 8: Gene Ontology analysis of genes differentially expressed in BC-PyMT *vs* HBCx-5, HBCx-24 and HBCx-34 TAMs.

BC-PyMT vs HBCx-5

Term Type	GO ID with Link	Go Term	Nb Regulated Genes (Up / Down)	P-Value
biological_process	GO:0006955	immune response	41 (25/16)	2,43E-11
biological_process	GO:0002376	immune system process	54 (31/23)	1,01E-10
cellular_component	GO:0005773	vacuole	26 (5/21)	6,18E-10
cellular_component	GO:0005764	lysosome	24 (5/19)	1,05E-09
cellular_component	GO:0000323	lytic vacuole	24 (5/19)	1,18E-09
biological_process	GO:0006950	response to stress	62 (27/35)	9,35E-08
cellular_component	GO:0005737	cytoplasm	232 (66/166)	3,89E-07
biological_process	GO:0050896	response to stimulus	101 (49/52)	4,20E-07
biological_process	GO:0006952	defense response	32 (17/15)	5,15E-07
biological_process	GO:0006954	inflammatory response	21 (11/10)	1,38E-06
cellular_component	GO:0044444	cytoplasmic part	163 (39/124)	1,58E-06
biological_process	GO:0009607	response to biotic stimulus	25 (17/8)	1,88E-06
biological_process	GO:0051707	response to other organism	21 (13/8)	3,09E-06
biological_process	GO:0048584	positive regulation of response to stimulus	18 (8/10)	5,91E-06
molecular_function	GO:0003824	catalytic activity	176 (53/123)	9,00E-06
biological_process	GO:0048583	regulation of response to stimulus	24 (9/15)	1,41E-05
biological_process	GO:0009615	response to virus	11 (7/4)	2,27E-05
biological_process	GO:0080134	regulation of response to stress	17 (6/11)	2,71E-05
cellular_component	GO:0005768	endosome	21 (4/17)	3,07E-05
biological_process	GO:0012501	programmed cell death	29 (12/17)	3,43E-05
biological_process	GO:0008219	cell death	30 (13/17)	4,69E-05
biological_process	GO:0048522	positive regulation of cellular process	58 (27/31)	5,81E-05
biological_process	GO:0006915	apoptotic process	28 (11/17)	6,58E-05
biological_process	GO:0016265	death	30 (13/17)	7,05E-05
molecular_function	GO:0016787	hydrolase activity	87 (30/57)	7,11E-05
biological_process	GO:0048518	positive regulation of biological process	63 (28/35)	8,15E-05
biological_process	GO:0009611	response to wounding	23 (11/12)	8,84E-05
biological_process	GO:0006629	lipid metabolic process	36 (5/31)	1,06E-04
biological_process	GO:0051704	multi-organism process	23 (13/10)	1,39E-04
molecular_function	GO:0003924	GTPase activity	13 (9/4)	1,44E-04
biological_process	GO:0042127	regulation of cell proliferation	29 (12/17)	3,08E-04
biological_process	GO:0046519	sphingoid metabolic process	7 (0/7)	3,73E-04
biological_process	GO:0002703	regulation of leukocyte mediated immunity	9 (4/5)	4,04E-04
biological_process	GO:0050776	regulation of immune response	15 (6/9)	4,08E-04
biological_process	GO:0002697	regulation of immune effector process	10 (4/6)	4,12E-04
biological_process	GO:0009987	cellular process	267 (96/171)	5,78E-04
cellular_component	GO:0048770	pigment granule	10 (2/8)	6,22E-04
cellular_component	GO:0042470	melanosome	10 (2/8)	6,22E-04
molecular_function	GO:0030246	carbohydrate binding	20 (7/13)	7,75E-04
molecular_function	GO:0005125	cytokine activity	14 (8/6)	9,86E-04
biological_process	GO:0006665	sphingolipid metabolic process	8 (0/8)	9,97E-04
cellular_component	GO:0043231	intracellular membrane-bounded organelle	235 (80/155)	1,07E-03
cellular_component	GO:0043227	membrane-bounded organelle	235 (80/155)	1,13E-03
biological_process	GO:0006643	membrane lipid metabolic process	8 (0/8)	1,21E-03
molecular_function	GO:0016788	hydrolase activity, acting on ester bonds	32 (9/23)	1,21E-03
biological_process	GO:0044255	cellular lipid metabolic process	25 (5/20)	1,48E-03
biological_process	GO:0051239	regulation of multicellular organismal process	35 (15/20)	1,64E-03
biological_process	GO:0002682	regulation of immune system process	19 (9/10)	1,65E-03
biological_process	GO:0008285	negative regulation of cell proliferation	15 (8/7)	1,92E-03
molecular_function	GO:0004416	hydroxyacylglutathione hydrolase activity	3 (0/3)	2,04E-03
biological_process	GO:0006672	ceramide metabolic process	6 (0/6)	2,10E-03
biological_process	GO:0009605	response to external stimulus	30 (15/15)	2,13E-03
biological_process	GO:0010627	regulation of intracellular protein kinase cascade	12 (5/7)	2,15E-03
biological_process	GO:0008152	metabolic process	207 (66/141)	2,20E-03
biological_process	GO:0002684	positive regulation of immune system process	14 (8/6)	2,53E-03
biological_process	GO:0050778	positive regulation of immune response	11 (5/6)	2,61E-03
biological_process	GO:0043408	regulation of MAPK cascade	9 (3/6)	2,62E-03
cellular_component	GO:0005615	extracellular space	27 (10/17)	2,64E-03
molecular_function	GO:0005164	tumor necrosis factor receptor binding	5 (2/3)	2,83E-03
molecular_function	GO:0008009	chemokine activity	6 (5/1)	3,04E-03
biological_process	GO:0001817	regulation of cytokine production	11 (4/7)	3,06E-03
molecular_function	GO:0005539	glycosaminoglycan binding	10 (4/6)	3,19E-03
biological_process	GO:0031349	positive regulation of defense response	7 (3/4)	3,26E-03
molecular_function	GO:0042379	chemokine receptor binding	6 (5/1)	3,42E-03
biological_process	GO:0045619	regulation of lymphocyte differentiation	7 (5/2)	3,56E-03
cellular_component	GO:0044424	intracellular part	296 (99/197)	3,56E-03
biological_process	GO:0044248	cellular catabolic process	36 (11/25)	3,57E-03
biological_process	GO:0006917	induction of apoptosis	12 (5/7)	3,83E-03
biological_process	GO:0012502	induction of programmed cell death	12 (5/7)	3,83E-03
biological_process	GO:0032879	regulation of localization	21 (8/13)	4,04E-03
biological_process	GO:0016192	vesicle-mediated transport	23 (6/17)	4,41E-03
molecular_function	GO:0032813	tumor necrosis factor receptor superfamily binding	5 (2/3)	4,49E-03
biological_process	GO:0031347	regulation of defense response	9 (3/6)	4,92E-03
biological_process	GO:0002252	immune effector process	10 (5/5)	5,09E-03
biological_process	GO:0048519	negative regulation of biological process	50 (20/30)	5,58E-03
cellular_component	GO:0009986	cell surface	19 (8/11)	5,89E-03
biological_process	GO:0050793	regulation of developmental process	26 (12/14)	6,07E-03
molecular_function	GO:0005525	GTP binding	19 (12/7)	6,16E-03
biological_process	GO:0045087	innate immune response	9 (4/5)	6,17E-03
biological_process	GO:0002699	positive regulation of immune effector process	6 (4/2)	6,20E-03
molecular_function	GO:0008201	heparin binding	8 (3/5)	6,25E-03

biological_process	GO:0002706	regulation of lymphocyte mediated immunity	7 (4/3)	6,26E-03
biological_process	GO:0002694	regulation of leukocyte activation	11 (6/5)	6,31E-03
biological_process	GO:0009056	catabolic process	41 (11/30)	6,42E-03
biological_process	GO:0008610	lipid biosynthetic process	16 (4/12)	6,72E-03
molecular_function	GO:0030247	polysaccharide binding	10 (4/6)	6,81E-03
molecular_function	GO:0001871	pattern binding	10 (4/6)	6,81E-03
biological_process	GO:0006979	response to oxidative stress	8 (0/8)	6,89E-03
biological_process	GO:0050865	regulation of cell activation	11 (6/5)	6,89E-03
cellular_component	GO:0016023	cytoplasmic membrane-bounded vesicle	22 (6/16)	6,93E-03
biological_process	GO:0045577	regulation of B cell differentiation	4 (3/1)	7,27E-03
cellular_component	GO:0005783	endoplasmic reticulum	37 (10/27)	7,46E-03
biological_process	GO:0055114	oxidation-reduction process	29 (3/26)	7,89E-03
molecular_function	GO:0032561	guanyl ribonucleotide binding	19 (12/7)	7,90E-03
molecular_function	GO:0019001	guanyl nucleotide binding	19 (12/7)	7,90E-03
cellular_component	GO:0031988	membrane-bounded vesicle	22 (6/16)	8,05E-03
biological_process	GO:0009408	response to heat	5 (4/1)	8,62E-03
biological_process	GO:0048523	negative regulation of cellular process	45 (18/27)	8,63E-03
biological_process	GO:0051716	cellular response to stimulus	25 (10/15)	8,90E-03
cellular_component	GO:0031410	cytoplasmic vesicle	25 (6/19)	9,27E-03
biological_process	GO:0009893	positive regulation of metabolic process	29 (12/17)	9,60E-03
biological_process	GO:0043123	positive regulation of I-kappaB kinase/NF-kappaB cascade	5 (3/2)	9,62E-03
molecular_function	GO:0016798	hydrolase activity, acting on glycosyl bonds	9 (0/9)	1,01E-02
biological_process	GO:0044237	cellular metabolic process	173 (63/110)	1,03E-02
biological_process	GO:0051251	positive regulation of lymphocyte activation	8 (5/3)	1,04E-02
cellular_component	GO:0005622	intracellular	304 (104/200)	1,06E-02
molecular_function	GO:0016667	oxidoreductase activity, acting on a sulfur group of donors	5 (1/4)	1,06E-02
biological_process	GO:0045621	positive regulation of lymphocyte differentiation	5 (4/1)	1,07E-02
biological_process	GO:0002521	leukocyte differentiation	10 (4/6)	1,08E-02
biological_process	GO:0006066	alcohol metabolic process	18 (3/15)	1,14E-02
biological_process	GO:0043065	positive regulation of apoptotic process	14 (7/7)	1,15E-02
cellular_component	GO:0031982	vesicle	25 (6/19)	1,18E-02
biological_process	GO:0051249	regulation of lymphocyte activation	10 (6/4)	1,18E-02
cellular_component	GO:0030173	integral to Golgi membrane	5 (0/5)	1,18E-02
cellular_component	GO:0031228	intrinsic to Golgi membrane	5 (0/5)	1,18E-02
biological_process	GO:0042221	response to chemical stimulus	35 (17/18)	1,23E-02
biological_process	GO:0080135	regulation of cellular response to stress	7 (3/4)	1,24E-02
cellular_component	GO:0005739	mitochondrion	52 (10/42)	1,24E-02
biological_process	GO:0043068	positive regulation of programmed cell death	14 (7/7)	1,24E-02
biological_process	GO:0050864	regulation of B cell activation	6 (3/3)	1,31E-02
biological_process	GO:0002449	lymphocyte mediated immunity	7 (4/3)	1,31E-02
biological_process	GO:0010942	positive regulation of cell death	14 (7/7)	1,31E-02
biological_process	GO:0009967	positive regulation of signal transduction	11 (6/5)	1,32E-02
molecular_function	GO:0005381	iron ion transmembrane transporter activity	3 (0/3)	1,33E-02
biological_process	GO:0002696	positive regulation of leukocyte activation	8 (5/3)	1,35E-02
cellular_component	GO:0009897	external side of plasma membrane	14 (7/7)	1,38E-02
biological_process	GO:0042552	myelination	5 (2/3)	1,43E-02
biological_process	GO:0000375	RNA splicing, via transesterification reactions	5 (3/2)	1,43E-02
biological_process	GO:0000398	nuclear mRNA splicing, via spliceosome	5 (3/2)	1,43E-02
biological_process	GO:0000377	RNA splicing, via transesterification reactions with bulged adeno	5 (3/2)	1,43E-02
biological_process	GO:0031325	positive regulation of cellular metabolic process	27 (12/15)	1,48E-02
biological_process	GO:0008284	positive regulation of cell proliferation	15 (5/10)	1,49E-02
biological_process	GO:0050867	positive regulation of cell activation	8 (5/3)	1,50E-02
biological_process	GO:0006518	peptide metabolic process	5 (0/5)	1,57E-02
biological_process	GO:0045579	positive regulation of B cell differentiation	3 (2/1)	1,65E-02
biological_process	GO:0032101	regulation of response to external stimulus	8 (3/5)	1,66E-02
biological_process	GO:0006873	cellular ion homeostasis	14 (5/9)	1,72E-02
biological_process	GO:0007272	ensheathment of neurons	5 (2/3)	1,72E-02
biological_process	GO:0008366	axon ensheathment	5 (2/3)	1,72E-02
biological_process	GO:0032103	positive regulation of response to external stimulus	5 (3/2)	1,72E-02
molecular_function	GO:0016860	intramolecular oxidoreductase activity	5 (1/4)	1,73E-02
cellular_component	GO:0043229	intracellular organelle	251 (87/164)	1,81E-02
molecular_function	GO:0005515	protein binding	176 (72/104)	1,82E-02
biological_process	GO:0051246	regulation of protein metabolic process	17 (2/15)	1,84E-02
biological_process	GO:0032844	regulation of homeostatic process	6 (3/3)	1,85E-02
cellular_component	GO:0043226	organelle	251 (87/164)	1,86E-02
biological_process	GO:0002708	positive regulation of lymphocyte mediated immunity	5 (4/1)	1,87E-02
biological_process	GO:0002705	positive regulation of leukocyte mediated immunity	5 (4/1)	1,87E-02
biological_process	GO:0043122	regulation of I-kappaB kinase/NF-kappaB cascade	5 (3/2)	1,87E-02
biological_process	GO:0060627	regulation of vesicle-mediated transport	7 (1/6)	1,96E-02
biological_process	GO:0009617	response to bacterium	10 (6/4)	1,98E-02
biological_process	GO:0044092	negative regulation of molecular function	9 (2/7)	2,02E-02
biological_process	GO:0051050	positive regulation of transport	9 (2/7)	2,02E-02
biological_process	GO:0055082	cellular chemical homeostasis	14 (5/9)	2,08E-02
biological_process	GO:0030316	osteoclast differentiation	3 (1/2)	2,09E-02
biological_process	GO:0006935	chemotaxis	8 (8/0)	2,19E-02
biological_process	GO:0042330	taxis	8 (8/0)	2,19E-02
biological_process	GO:0051247	positive regulation of protein metabolic process	8 (2/6)	2,19E-02
biological_process	GO:0016042	lipid catabolic process	9 (0/9)	2,19E-02
molecular_function	GO:0042802	identical protein binding	15 (5/10)	2,20E-02
biological_process	GO:0051094	positive regulation of developmental process	12 (7/5)	2,21E-02
biological_process	GO:0016064	immunoglobulin mediated immune response	6 (4/2)	2,24E-02
biological_process	GO:0010324	membrane invagination	11 (3/8)	2,30E-02

biological_process	GO:0006897	endocytosis	11 (3/8)	2,30E-02
biological_process	GO:0016044	cellular membrane organization	14 (4/10)	2,32E-02
biological_process	GO:0001568	blood vessel development	13 (3/10)	2,35E-02
biological_process	GO:0010647	positive regulation of cell communication	11 (6/5)	2,38E-02
molecular_function	GO:0046915	transition metal ion transmembrane transporter activity	4 (0/4)	2,44E-02
biological_process	GO:0019724	B cell mediated immunity	6 (4/2)	2,53E-02
biological_process	GO:0010740	positive regulation of intracellular protein kinase cascade	7 (4/3)	2,53E-02
biological_process	GO:0006684	sphingomyelin metabolic process	3 (0/3)	2,57E-02
biological_process	GO:0048878	chemical homeostasis	17 (6/11)	2,60E-02
biological_process	GO:0031399	regulation of protein modification process	10 (2/8)	2,62E-02
biological_process	GO:0002443	leukocyte mediated immunity	7 (4/3)	2,66E-02
biological_process	GO:0050870	positive regulation of T cell activation	6 (4/2)	2,68E-02
biological_process	GO:0042592	homeostatic process	24 (8/16)	2,70E-02
biological_process	GO:0030098	lymphocyte differentiation	8 (3/5)	2,72E-02
molecular_function	GO:0050840	extracellular matrix binding	4 (3/1)	2,72E-02
molecular_function	GO:0016491	oxidoreductase activity	29 (4/25)	2,73E-02
biological_process	GO:0042981	regulation of apoptotic process	23 (10/13)	2,75E-02
biological_process	GO:0019228	regulation of action potential in neuron	5 (2/3)	2,76E-02
biological_process	GO:0001944	vasculature development	13 (3/10)	2,77E-02
biological_process	GO:0055092	sterol homeostasis	4 (1/3)	2,80E-02
biological_process	GO:0042632	cholesterol homeostasis	4 (1/3)	2,80E-02
biological_process	GO:0030097	hemopoiesis	13 (5/8)	2,84E-02
biological_process	GO:0032268	regulation of cellular protein metabolic process	14 (2/12)	2,85E-02
cellular_component	GO:0005794	Golgi apparatus	29 (6/23)	2,91E-02
biological_process	GO:0051336	regulation of hydrolase activity	11 (3/8)	2,96E-02
cellular_component	GO:0044421	extracellular region part	32 (12/20)	2,96E-02
molecular_function	GO:0004364	glutathione transferase activity	4 (0/4)	3,02E-02
biological_process	GO:0019725	cellular homeostasis	16 (5/11)	3,06E-02
cellular_component	GO:0000267	cell fraction	26 (5/21)	3,06E-02
biological_process	GO:0044238	primary metabolic process	178 (62/116)	3,08E-02
biological_process	GO:0006041	glucosamine metabolic process	3 (0/3)	3,09E-02
biological_process	GO:0006044	N-acetylglucosamine metabolic process	3 (0/3)	3,09E-02
biological_process	GO:0048514	blood vessel morphogenesis	11 (2/9)	3,14E-02
biological_process	GO:0033554	cellular response to stress	18 (6/12)	3,14E-02
biological_process	GO:0043067	regulation of programmed cell death	23 (10/13)	3,17E-02
cellular_component	GO:0045121	membrane raft	7 (1/6)	3,22E-02
molecular_function	GO:0019865	immunoglobulin binding	3 (1/2)	3,26E-02
biological_process	GO:0010941	regulation of cell death	23 (10/13)	3,27E-02
biological_process	GO:0051049	regulation of transport	14 (4/10)	3,30E-02
molecular_function	GO:0004553	hydrolase activity, hydrolyzing O-glycosyl compounds	7 (0/7)	3,31E-02
biological_process	GO:0008203	cholesterol metabolic process	6 (1/5)	3,34E-02
biological_process	GO:0070201	regulation of establishment of protein localization	6 (1/5)	3,52E-02
biological_process	GO:0006909	phagocytosis	5 (2/3)	3,63E-02
biological_process	GO:0002237	response to molecule of bacterial origin	5 (3/2)	3,63E-02
biological_process	GO:0045597	positive regulation of cell differentiation	10 (6/4)	3,63E-02
biological_process	GO:0010829	negative regulation of glucose transport	3 (2/1)	3,64E-02
biological_process	GO:0046513	ceramide biosynthetic process	3 (0/3)	3,64E-02
molecular_function	GO:0005102	receptor binding	27 (12/15)	3,66E-02
biological_process	GO:0051259	protein oligomerization	6 (1/5)	3,71E-02
biological_process	GO:0002761	regulation of myeloid leukocyte differentiation	4 (1/3)	3,72E-02
biological_process	GO:0051054	positive regulation of DNA metabolic process	4 (2/2)	3,72E-02
molecular_function	GO:0001664	G-protein coupled receptor binding	6 (5/1)	3,72E-02
biological_process	GO:0051130	positive regulation of cellular component organization	8 (4/4)	3,73E-02
biological_process	GO:0010564	regulation of cell cycle process	6 (4/2)	3,90E-02
biological_process	GO:0050801	ion homeostasis	14 (5/9)	3,91E-02
cellular_component	GO:0031301	integral to organelle membrane	7 (1/6)	3,91E-02
biological_process	GO:0032502	developmental process	85 (35/50)	3,91E-02
cellular_component	GO:0000139	Golgi membrane	9 (0/9)	3,94E-02
biological_process	GO:0045595	regulation of cell differentiation	17 (7/10)	4,03E-02
biological_process	GO:0002700	regulation of production of molecular mediator of immune respor	4 (0/4)	4,06E-02
biological_process	GO:0065009	regulation of molecular function	22 (5/17)	4,06E-02
biological_process	GO:0009266	response to temperature stimulus	5 (4/1)	4,11E-02
biological_process	GO:0045637	regulation of myeloid cell differentiation	5 (1/4)	4,11E-02
biological_process	GO:0030149	sphingolipid catabolic process	3 (0/3)	4,23E-02
biological_process	GO:0046520	sphingoid biosynthetic process	3 (0/3)	4,23E-02
biological_process	GO:0019915	lipid storage	3 (0/3)	4,23E-02
biological_process	GO:0046466	membrane lipid catabolic process	3 (0/3)	4,23E-02
biological_process	GO:0032270	positive regulation of cellular protein metabolic process	7 (2/5)	4,33E-02
biological_process	GO:0005975	carbohydrate metabolic process	19 (2/17)	4,46E-02
biological_process	GO:0043086	negative regulation of catalytic activity	7 (2/5)	4,51E-02
biological_process	GO:0030278	regulation of ossification	5 (4/1)	4,64E-02
biological_process	GO:0051260	protein homooligomerization	5 (1/4)	4,64E-02
biological_process	GO:0002695	negative regulation of leukocyte activation	5 (3/2)	4,64E-02
biological_process	GO:0050866	negative regulation of cell activation	5 (3/2)	4,64E-02
cellular_component	GO:0005902	microvillus	4 (2/2)	4,68E-02
biological_process	GO:0016125	sterol metabolic process	6 (1/5)	4,73E-02
biological_process	GO:0045582	positive regulation of T cell differentiation	4 (3/1)	4,77E-02
biological_process	GO:0060191	regulation of lipase activity	4 (0/4)	4,77E-02
biological_process	GO:0006826	iron ion transport	4 (0/4)	4,77E-02
biological_process	GO:0045669	positive regulation of osteoblast differentiation	3 (2/1)	4,85E-02
biological_process	GO:0045739	positive regulation of DNA repair	3 (2/1)	4,85E-02
biological_process	GO:0051726	regulation of cell cycle	11 (6/5)	4,90E-02

biological_process	GO:0001508	regulation of action potential	5 (2/3)	4,91E-02
biological_process	GO:0031401	positive regulation of protein modification process	6 (2/4)	4,95E-02
biological_process	GO:0007275	multicellular organismal development	78 (31/47)	5,04E-02
biological_process	GO:0050790	regulation of catalytic activity	19 (5/14)	5,12E-02
biological_process	GO:0006029	proteoglycan metabolic process	4 (1/3)	5,16E-02
molecular_function	GO:0004342	glucosamine-6-phosphate deaminase activity	2 (0/2)	5,20E-02
biological_process	GO:0001775	cell activation	12 (5/7)	5,22E-02
biological_process	GO:0032944	regulation of mononuclear cell proliferation	6 (4/2)	5,42E-02
biological_process	GO:0050670	regulation of lymphocyte proliferation	6 (4/2)	5,42E-02
biological_process	GO:0019221	cytokine-mediated signaling pathway	5 (4/1)	5,48E-02
biological_process	GO:0002819	regulation of adaptive immune response	5 (3/2)	5,48E-02
biological_process	GO:0002822	regulation of adaptive immune response based on somatic recor	5 (3/2)	5,48E-02
biological_process	GO:0002886	regulation of myeloid leukocyte mediated immunity	3 (1/2)	5,51E-02
biological_process	GO:0006911	phagocytosis, engulfment	3 (1/2)	5,51E-02
biological_process	GO:0051607	defense response to virus	3 (1/2)	5,51E-02
biological_process	GO:0002824	positive regulation of adaptive immune response based on soma	4 (3/1)	5,55E-02
biological_process	GO:0055088	lipid homeostasis	4 (1/3)	5,55E-02
biological_process	GO:0002573	myeloid leukocyte differentiation	4 (2/2)	5,55E-02
biological_process	GO:0002821	positive regulation of adaptive immune response	4 (3/1)	5,55E-02
biological_process	GO:0048856	anatomical structure development	68 (27/41)	5,56E-02
biological_process	GO:0048731	system development	64 (24/40)	5,62E-02
biological_process	GO:0006633	fatty acid biosynthetic process	6 (2/4)	5,66E-02
biological_process	GO:0030005	cellular di-, tri-valent inorganic cation homeostasis	8 (3/5)	5,67E-02
cellular_component	GO:0044431	Golgi apparatus part	12 (1/11)	5,70E-02
biological_process	GO:0005996	monosaccharide metabolic process	10 (1/9)	5,74E-02
biological_process	GO:0048534	hemopoietic or lymphoid organ development	13 (5/8)	5,76E-02
molecular_function	GO:0017111	nucleoside-triphosphatase activity	24 (14/10)	5,78E-02
biological_process	GO:0050727	regulation of inflammatory response	5 (2/3)	5,78E-02
biological_process	GO:0010604	positive regulation of macromolecule metabolic process	24 (10/14)	5,88E-02
biological_process	GO:0050863	regulation of T cell activation	7 (4/3)	5,89E-02
biological_process	GO:0070663	regulation of leukocyte proliferation	6 (4/2)	5,91E-02
biological_process	GO:0032945	negative regulation of mononuclear cell proliferation	4 (3/1)	5,96E-02
biological_process	GO:0050672	negative regulation of lymphocyte proliferation	4 (3/1)	5,96E-02
biological_process	GO:0031214	biomineral tissue development	4 (2/2)	5,96E-02
biological_process	GO:0070664	negative regulation of leukocyte proliferation	4 (3/1)	5,96E-02
biological_process	GO:0016043	cellular component organization	60 (23/37)	5,99E-02
cellular_component	GO:0005625	soluble fraction	7 (2/5)	6,00E-02
biological_process	GO:0051052	regulation of DNA metabolic process	5 (2/3)	6,09E-02
biological_process	GO:0019216	regulation of lipid metabolic process	5 (1/4)	6,09E-02
biological_process	GO:0051783	regulation of nuclear division	4 (2/2)	6,38E-02
biological_process	GO:0045089	positive regulation of innate immune response	4 (1/3)	6,38E-02
biological_process	GO:0007204	elevation of cytosolic calcium ion concentration	4 (3/1)	6,38E-02
biological_process	GO:0051222	positive regulation of protein transport	4 (1/3)	6,38E-02
biological_process	GO:0007088	regulation of mitosis	4 (2/2)	6,38E-02
biological_process	GO:0000302	response to reactive oxygen species	4 (0/4)	6,38E-02
biological_process	GO:0002460	adaptive immune response based on somatic recombination of i	6 (4/2)	6,42E-02
biological_process	GO:0002250	adaptive immune response	6 (4/2)	6,42E-02
molecular_function	GO:0016817	hydrolase activity, acting on acid anhydrides	25 (15/10)	6,44E-02
cellular_component	GO:0005637	nuclear inner membrane	3 (2/1)	6,49E-02
molecular_function	GO:0015929	hexosaminidase activity	3 (0/3)	6,52E-02
biological_process	GO:0030324	lung development	7 (1/6)	6,55E-02
molecular_function	GO:0004091	carboxylesterase activity	7 (0/7)	6,60E-02
biological_process	GO:0042327	positive regulation of phosphorylation	5 (2/3)	6,74E-02
molecular_function	GO:0005488	binding	315 (134/181)	6,81E-02
biological_process	GO:0050871	positive regulation of B cell activation	4 (2/2)	6,82E-02
cellular_component	GO:0031225	anchored to membrane	12 (6/6)	6,87E-02
biological_process	GO:0006282	regulation of DNA repair	3 (2/1)	6,90E-02
biological_process	GO:0045670	regulation of osteoclast differentiation	3 (1/2)	6,90E-02
biological_process	GO:0050869	negative regulation of B cell activation	3 (2/1)	6,90E-02
biological_process	GO:0030323	respiratory tube development	7 (1/6)	7,02E-02
biological_process	GO:0042325	regulation of phosphorylation	13 (4/9)	7,03E-02
biological_process	GO:0016053	organic acid biosynthetic process	8 (3/5)	7,05E-02
biological_process	GO:0046394	carboxylic acid biosynthetic process	8 (3/5)	7,05E-02
molecular_function	GO:0016298	lipase activity	6 (0/6)	7,09E-02
biological_process	GO:0051480	cytosolic calcium ion homeostasis	4 (3/1)	7,27E-02
molecular_function	GO:0019900	kinase binding	6 (3/3)	7,38E-02
biological_process	GO:0010562	positive regulation of phosphorus metabolic process	5 (2/3)	7,41E-02
biological_process	GO:0045937	positive regulation of phosphate metabolic process	5 (2/3)	7,41E-02
cellular_component	GO:0044440	endosomal part	4 (2/2)	7,42E-02
cellular_component	GO:0010008	endosome membrane	4 (2/2)	7,42E-02
biological_process	GO:0006686	sphingomyelin biosynthetic process	2 (0/2)	7,49E-02
biological_process	GO:0010744	positive regulation of macrophage derived foam cell differentiatio	2 (0/2)	7,49E-02
biological_process	GO:0034614	cellular response to reactive oxygen species	3 (0/3)	7,64E-02
molecular_function	GO:0030911	TPR domain binding	2 (1/1)	7,70E-02
biological_process	GO:0002520	immune system development	13 (5/8)	7,75E-02
biological_process	GO:0035295	tube development	12 (3/9)	7,76E-02
biological_process	GO:0044262	cellular carbohydrate metabolic process	14 (1/13)	7,84E-02
molecular_function	GO:0016780	phosphotransferase activity, for other substituted phosphate grou	3 (1/2)	8,03E-02
biological_process	GO:0032880	regulation of protein localization	6 (1/5)	8,10E-02
biological_process	GO:0042035	regulation of cytokine biosynthetic process	5 (2/3)	8,12E-02
biological_process	GO:0001666	response to hypoxia	5 (1/4)	8,12E-02
biological_process	GO:0000003	reproduction	23 (5/18)	8,14E-02

biological_process	GO:0055066	di-, tri-valent inorganic cation homeostasis	8 (3/5)	8,15E-02
biological_process	GO:0007243	intracellular protein kinase cascade	11 (4/7)	8,24E-02
molecular_function	GO:0016462	pyrophosphatase activity	24 (14/10)	8,24E-02
biological_process	GO:0045428	regulation of nitric oxide biosynthetic process	3 (2/1)	8,40E-02
biological_process	GO:0006040	amino sugar metabolic process	3 (0/3)	8,40E-02
biological_process	GO:0006874	cellular calcium ion homeostasis	6 (3/3)	8,40E-02
biological_process	GO:0009966	regulation of signal transduction	24 (8/16)	8,43E-02
biological_process	GO:0051223	regulation of protein transport	5 (1/4)	8,49E-02
biological_process	GO:0070482	response to oxygen levels	5 (1/4)	8,49E-02
biological_process	GO:0042391	regulation of membrane potential	7 (2/5)	8,52E-02
molecular_function	GO:0016818	hydrolase activity, acting on acid anhydrides, in phosphorus-con	24 (14/10)	8,64E-02
biological_process	GO:0019220	regulation of phosphate metabolic process	13 (4/9)	8,68E-02
biological_process	GO:0051174	regulation of phosphorus metabolic process	13 (4/9)	8,68E-02
biological_process	GO:0007626	locomotory behavior	11 (9/2)	8,77E-02
biological_process	GO:0043066	negative regulation of apoptotic process	11 (3/8)	8,77E-02
biological_process	GO:0043933	macromolecular complex subunit organization	15 (5/10)	8,92E-02
biological_process	GO:0001932	regulation of protein phosphorylation	7 (2/5)	9,05E-02
biological_process	GO:0010827	regulation of glucose transport	3 (2/1)	9,18E-02
biological_process	GO:0042108	positive regulation of cytokine biosynthetic process	4 (1/3)	9,20E-02
biological_process	GO:0044270	cellular nitrogen compound catabolic process	4 (3/1)	9,20E-02
biological_process	GO:0009892	negative regulation of metabolic process	20 (6/14)	9,28E-02
biological_process	GO:0006790	sulfur compound metabolic process	6 (1/5)	9,34E-02
biological_process	GO:0030155	regulation of cell adhesion	6 (4/2)	9,34E-02
biological_process	GO:0065003	macromolecular complex assembly	14 (5/9)	9,34E-02
cellular_component	GO:0031300	intrinsic to organelle membrane	7 (1/6)	9,54E-02
biological_process	GO:0030003	cellular cation homeostasis	8 (3/5)	9,58E-02
biological_process	GO:0055074	calcium ion homeostasis	6 (3/3)	9,66E-02
biological_process	GO:0050900	leukocyte migration	4 (3/1)	9,71E-02
biological_process	GO:0045785	positive regulation of cell adhesion	4 (3/1)	9,71E-02
biological_process	GO:0001816	cytokine production	4 (1/3)	9,71E-02
biological_process	GO:0043069	negative regulation of programmed cell death	11 (3/8)	9,72E-02
biological_process	GO:0045416	positive regulation of interleukin-8 biosynthetic process	2 (0/2)	9,85E-02
biological_process	GO:0045414	regulation of interleukin-8 biosynthetic process	2 (0/2)	9,85E-02
biological_process	GO:0010743	regulation of macrophage derived foam cell differentiation	2 (0/2)	9,85E-02
biological_process	GO:0032288	myelin assembly	2 (0/2)	9,85E-02
biological_process	GO:0033209	tumor necrosis factor-mediated signaling pathway	2 (1/1)	9,85E-02
biological_process	GO:0032677	regulation of interleukin-8 production	2 (0/2)	9,85E-02
biological_process	GO:0010033	response to organic substance	19 (9/10)	9,86E-02
cellular_component	GO:0005576	extracellular region	56 (27/29)	9,86E-02
biological_process	GO:0060541	respiratory system development	7 (1/6)	9,89E-02
biological_process	GO:0031396	regulation of protein ubiquitination	3 (0/3)	9,98E-02
biological_process	GO:0030148	sphingolipid biosynthetic process	3 (0/3)	9,98E-02
biological_process	GO:0045638	negative regulation of myeloid cell differentiation	3 (1/2)	9,98E-02
biological_process	GO:0006446	regulation of translational initiation	3 (0/3)	9,98E-02

BC-PyMT *vs* HBCx-24

Term Type	GO ID with Link	Go Term	Nb Regulated Genes (Up / Down)	P-Value
biological_process	GO:0002376	immune system process	40 (29/11)	2,93E-07
cellular_component	GO:0005622	intracellular	248 (167/81)	4,03E-06
cellular_component	GO:0044424	intracellular part	236 (155/81)	2,82E-05
cellular_component	GO:0016604	nuclear body	13 (10/3)	4,19E-05
molecular_function	GO:0005488	binding	259 (191/68)	6,42E-05
biological_process	GO:0009615	response to virus	9 (7/2)	1,58E-04
biological_process	GO:0009987	cellular process	218 (147/71)	1,71E-04
biological_process	GO:0051246	regulation of protein metabolic process	19 (14/5)	3,37E-04
biological_process	GO:0044237	cellular metabolic process	149 (104/45)	4,82E-04
biological_process	GO:0006955	immune response	22 (17/5)	7,10E-04
cellular_component	GO:0005737	cytoplasm	164 (101/63)	8,65E-04
biological_process	GO:0019221	cytokine-mediated signaling pathway	7 (6/1)	9,60E-04
cellular_component	GO:0043227	membrane-bounded organelle	180 (116/64)	1,14E-03
cellular_component	GO:0043229	intracellular organelle	199 (129/70)	1,22E-03
cellular_component	GO:0043226	organelle	199 (129/70)	1,26E-03
biological_process	GO:0032268	regulation of cellular protein metabolic process	15 (11/4)	1,90E-03
biological_process	GO:0009607	response to biotic stimulus	16 (12/4)	2,08E-03
cellular_component	GO:0043231	intracellular membrane-bounded organelle	178 (114/64)	2,25E-03
cellular_component	GO:0005829	cytosol	23 (15/8)	2,65E-03
cellular_component	GO:0016607	nuclear speck	8 (6/2)	2,71E-03
molecular_function	GO:0005515	protein binding	143 (105/38)	3,30E-03
biological_process	GO:0051707	response to other organism	13 (9/4)	3,59E-03
biological_process	GO:0030500	regulation of bone mineralization	4 (2/2)	3,90E-03
biological_process	GO:0070167	regulation of biomineral tissue development	4 (2/2)	3,90E-03
molecular_function	GO:0003924	GTPase activity	9 (8/1)	4,28E-03
biological_process	GO:0030705	cytoskeleton-dependent intracellular transport	5 (5/0)	4,41E-03
cellular_component	GO:0005938	cell cortex	9 (7/2)	4,86E-03
biological_process	GO:0048534	hemopoietic or lymphoid organ development	14 (11/3)	5,32E-03
molecular_function	GO:0005525	GTP binding	16 (13/3)	5,39E-03
molecular_function	GO:0019001	guanyl nucleotide binding	16 (13/3)	6,72E-03
molecular_function	GO:0032561	guanyl ribonucleotide binding	16 (13/3)	6,72E-03
cellular_component	GO:0016605	PML body	4 (3/1)	6,76E-03
biological_process	GO:0022402	cell cycle process	17 (13/4)	7,10E-03
biological_process	GO:0006915	apoptotic process	19 (17/2)	7,33E-03
biological_process	GO:0044260	cellular macromolecule metabolic process	114 (81/33)	7,52E-03
cellular_component	GO:0005634	nucleus	111 (81/30)	7,75E-03
biological_process	GO:0002520	immune system development	14 (11/3)	7,87E-03
cellular_component	GO:0044444	cytoplasmic part	108 (58/50)	8,01E-03
cellular_component	GO:0030529	ribonucleoprotein complex	19 (6/13)	8,30E-03
biological_process	GO:0008219	cell death	20 (17/3)	8,47E-03
biological_process	GO:0012501	programmed cell death	19 (17/2)	8,69E-03
biological_process	GO:0016265	death	20 (17/3)	1,06E-02
biological_process	GO:0010608	posttranscriptional regulation of gene expression	9 (5/4)	1,12E-02
molecular_function	GO:0003735	structural constituent of ribosome	9 (0/9)	1,12E-02
cellular_component	GO:0070469	respiratory chain	6 (0/6)	1,13E-02
molecular_function	GO:0050136	NADH dehydrogenase (quinone) activity	4 (0/4)	1,18E-02
molecular_function	GO:0003954	NADH dehydrogenase activity	4 (0/4)	1,18E-02
molecular_function	GO:0008137	NADH dehydrogenase (ubiquinone) activity	4 (0/4)	1,18E-02
biological_process	GO:0050896	response to stimulus	66 (50/16)	1,26E-02
biological_process	GO:0044267	cellular protein metabolic process	58 (39/19)	1,28E-02
biological_process	GO:0030099	myeloid cell differentiation	7 (5/2)	1,39E-02
biological_process	GO:0006412	translation	14 (2/12)	1,43E-02
biological_process	GO:0045321	leukocyte activation	11 (9/2)	1,49E-02
molecular_function	GO:0032555	purine ribonucleotide binding	50 (46/4)	1,54E-02
molecular_function	GO:0032553	ribonucleotide binding	50 (46/4)	1,54E-02
molecular_function	GO:0019904	protein domain specific binding	10 (7/3)	1,55E-02
molecular_function	GO:0016655	oxidoreductase activity, acting on NADH or NADPH, qu	4 (0/4)	1,64E-02
biological_process	GO:0006417	regulation of translation	7 (4/3)	1,68E-02
biological_process	GO:0046649	lymphocyte activation	10 (8/2)	1,69E-02
cellular_component	GO:0044428	nuclear part	38 (27/11)	1,71E-02
biological_process	GO:0042592	homeostatic process	21 (12/9)	1,71E-02
biological_process	GO:0008285	negative regulation of cell proliferation	11 (8/3)	1,72E-02
cellular_component	GO:0034361	very-low-density lipoprotein particle	2 (1/1)	1,74E-02
cellular_component	GO:0034385	triglyceride-rich lipoprotein particle	2 (1/1)	1,74E-02
cellular_component	GO:0034358	plasma lipoprotein particle	3 (1/2)	1,81E-02
cellular_component	GO:0032994	protein-lipid complex	3 (1/2)	1,81E-02
cellular_component	GO:0005840	ribosome	10 (0/10)	1,93E-02
biological_process	GO:0031399	regulation of protein modification process	9 (8/1)	2,03E-02
molecular_function	GO:0017076	purine nucleotide binding	51 (47/4)	2,06E-02
biological_process	GO:0006996	organelle organization	34 (26/8)	2,14E-02
cellular_component	GO:0032991	macromolecular complex	68 (31/37)	2,25E-02
biological_process	GO:0002260	lymphocyte homeostasis	4 (2/2)	2,29E-02
biological_process	GO:0007163	establishment or maintenance of cell polarity	4 (4/0)	2,50E-02
biological_process	GO:0044265	cellular macromolecule catabolic process	21 (17/4)	2,57E-02
molecular_function	GO:0000166	nucleotide binding	57 (50/7)	2,98E-02
biological_process	GO:0001775	cell activation	11 (9/2)	3,00E-02
biological_process	GO:0051301	cell division	12 (8/4)	3,03E-02
biological_process	GO:0046907	intracellular transport	16 (12/4)	3,19E-02
cellular_component	GO:0042470	melanosome	6 (3/3)	3,22E-02
cellular_component	GO:0048770	pigment granule	6 (3/3)	3,22E-02
cellular_component	GO:0000502	proteasome complex	5 (2/3)	3,25E-02

molecular_function	GO:0042802	identical protein binding	12 (11/1)	3,28E-02
biological_process	GO:0051704	multi-organism process	14 (10/4)	3,32E-02
biological_process	GO:0030097	hemopoiesis	11 (9/2)	3,41E-02
biological_process	GO:0031214	biomineral tissue development	4 (1/3)	3,43E-02
biological_process	GO:0030048	actin filament-based movement	3 (3/0)	3,68E-02
cellular_component	GO:0044451	nucleoplasm part	18 (12/6)	4,03E-02
biological_process	GO:0001768	establishment of T cell polarity	2 (2/0)	4,05E-02
biological_process	GO:0001767	establishment of lymphocyte polarity	2 (2/0)	4,05E-02
molecular_function	GO:0016817	hydrolase activity, acting on acid anhydrides	21 (20/1)	4,08E-02
molecular_function	GO:0046983	protein dimerization activity	13 (12/1)	4,22E-02
biological_process	GO:0044248	cellular catabolic process	26 (21/5)	4,23E-02
biological_process	GO:0006397	mRNA processing	11 (7/4)	4,36E-02
biological_process	GO:0001776	leukocyte homeostasis	4 (2/2)	4,51E-02
molecular_function	GO:0004715	non-membrane spanning protein tyrosine kinase activi	4 (4/0)	4,58E-02
molecular_function	GO:0008289	lipid binding	13 (12/1)	4,59E-02
biological_process	GO:0007049	cell cycle	20 (15/5)	4,63E-02
biological_process	GO:0016071	mRNA metabolic process	12 (8/4)	4,68E-02
biological_process	GO:0016043	cellular component organization	51 (39/12)	4,70E-02
biological_process	GO:0009057	macromolecule catabolic process	21 (17/4)	4,72E-02
biological_process	GO:0051603	proteolysis involved in cellular protein catabolic proce:	18 (15/3)	4,82E-02
biological_process	GO:0033036	macromolecule localization	26 (17/9)	4,90E-02
biological_process	GO:0044257	cellular protein catabolic process	18 (15/3)	5,01E-02
biological_process	GO:0008152	metabolic process	159 (111/48)	5,06E-02
biological_process	GO:0051641	cellular localization	21 (15/6)	5,21E-02
biological_process	GO:0008380	RNA splicing	9 (5/4)	5,50E-02
biological_process	GO:0009056	catabolic process	30 (23/7)	5,59E-02
cellular_component	GO:0044422	organelle part	81 (52/29)	5,69E-02
biological_process	GO:0030163	protein catabolic process	18 (15/3)	6,51E-02
biological_process	GO:0000279	M phase	11 (8/3)	6,63E-02
biological_process	GO:0022607	cellular component assembly	18 (11/7)	6,72E-02
biological_process	GO:0030029	actin filament-based process	8 (8/0)	7,02E-02
biological_process	GO:0043170	macromolecule metabolic process	118 (84/34)	7,14E-02
biological_process	GO:0002521	leukocyte differentiation	7 (7/0)	7,17E-02
biological_process	GO:0042330	taxis	6 (5/1)	7,31E-02
biological_process	GO:0006935	chemotaxis	6 (5/1)	7,31E-02
molecular_function	GO:0017124	SH3 domain binding	5 (4/1)	7,43E-02
molecular_function	GO:0051015	actin filament binding	4 (4/0)	7,50E-02
biological_process	GO:0022403	cell cycle phase	12 (9/3)	7,50E-02
biological_process	GO:0043933	macromolecular complex subunit organization	14 (8/6)	7,55E-02
biological_process	GO:0050793	regulation of developmental process	18 (14/4)	7,56E-02
biological_process	GO:0051248	negative regulation of protein metabolic process	5 (4/1)	7,58E-02
cellular_component	GO:0005654	nucleoplasm	19 (13/6)	7,64E-02
biological_process	GO:0051649	establishment of localization in cell	19 (13/6)	7,75E-02
molecular_function	GO:0051536	iron-sulfur cluster binding	4 (2/2)	7,87E-02
molecular_function	GO:0051540	metal cluster binding	4 (2/2)	7,87E-02
biological_process	GO:0033209	tumor necrosis factor-mediated signaling pathway	2 (2/0)	7,94E-02
biological_process	GO:0031063	regulation of histone deacetylation	2 (2/0)	7,94E-02
biological_process	GO:0032020	ISG15-protein conjugation	2 (2/0)	7,94E-02
biological_process	GO:0031065	positive regulation of histone deacetylation	2 (2/0)	7,94E-02
biological_process	GO:0009186	deoxyribonucleoside diphosphate metabolic process	2 (2/0)	7,94E-02
biological_process	GO:0046631	alpha-beta T cell activation	3 (2/1)	7,97E-02
biological_process	GO:0022900	electron transport chain	6 (0/6)	8,00E-02
molecular_function	GO:0005083	small GTPase regulator activity	9 (9/0)	8,14E-02
cellular_component	GO:0044455	mitochondrial membrane part	4 (1/3)	8,18E-02
biological_process	GO:0048872	homeostasis of number of cells	6 (3/3)	8,24E-02
biological_process	GO:0009894	regulation of catabolic process	4 (2/2)	8,24E-02
molecular_function	GO:0016651	oxidoreductase activity, acting on NADH or NADPH	4 (0/4)	8,24E-02
cellular_component	GO:0044446	intracellular organelle part	79 (50/29)	8,40E-02
molecular_function	GO:0003824	catalytic activity	115 (92/23)	8,41E-02
molecular_function	GO:0005506	iron ion binding	12 (9/3)	8,62E-02
biological_process	GO:0048513	organ development	43 (34/9)	8,71E-02
biological_process	GO:0044238	primary metabolic process	140 (102/38)	8,72E-02
biological_process	GO:0065003	macromolecular complex assembly	13 (7/6)	8,81E-02
biological_process	GO:0042110	T cell activation	6 (5/1)	8,97E-02
molecular_function	GO:0030234	enzyme regulator activity	21 (19/2)	8,98E-02
molecular_function	GO:0005543	phospholipid binding	6 (5/1)	9,14E-02
molecular_function	GO:0016462	pyrophosphatase activity	19 (18/1)	9,42E-02
biological_process	GO:0030278	regulation of ossification	4 (2/2)	9,43E-02
biological_process	GO:0000280	nuclear division	8 (5/3)	9,44E-02
biological_process	GO:0007067	mitosis	8 (5/3)	9,44E-02
molecular_function	GO:0042803	protein homodimerization activity	8 (7/1)	9,50E-02
molecular_function	GO:0016879	ligase activity, forming carbon-nitrogen bonds	8 (7/1)	9,50E-02
biological_process	GO:0006461	protein complex assembly	9 (6/3)	9,52E-02
biological_process	GO:0070271	protein complex biogenesis	9 (6/3)	9,52E-02
molecular_function	GO:0016725	oxidoreductase activity, acting on CH or CH2 groups	2 (2/0)	9,64E-02
biological_process	GO:0050776	regulation of immune response	8 (6/2)	9,78E-02
molecular_function	GO:0016818	hydrolase activity, acting on acid anhydrides, in phospl	19 (18/1)	9,80E-02
cellular_component	GO:0005694	chromosome	14 (10/4)	9,81E-02
biological_process	GO:0000184	nuclear-transcribed mRNA catabolic process, nonsense	3 (2/1)	9,84E-02
biological_process	GO:0050777	negative regulation of immune response	3 (2/1)	9,84E-02

BC-PyMT vs HBCx-34

Term Type	GO ID with Link	Go Term	Nb Regulated Genes (Up / Down)	P-Value
biological_process	GO:0006955	immune response	31 (24/7)	1,26E-08
biological_process	GO:0002376	immune system process	40 (31/9)	8,67E-08
biological_process	GO:0009615	response to virus	11 (9/2)	2,13E-06
molecular_function	GO:0017076	purine nucleotide binding	64 (55/9)	2,24E-06
molecular_function	GO:0032553	ribonucleotide binding	62 (53/9)	2,49E-06
molecular_function	GO:0032555	purine ribonucleotide binding	62 (53/9)	2,49E-06
biological_process	GO:0045087	innate immune response	12 (10/2)	7,70E-06
cellular_component	GO:0044444	cytoplasmic part	120 (89/31)	8,39E-06
molecular_function	GO:0000166	nucleotide binding	69 (60/9)	1,29E-05
biological_process	GO:0009607	response to biotic stimulus	20 (17/3)	1,36E-05
biological_process	GO:0051707	response to other organism	16 (13/3)	6,73E-05
biological_process	GO:0006952	defense response	23 (16/7)	7,16E-05
cellular_component	GO:0044424	intracellular part	225 (160/65)	7,92E-05
molecular_function	GO:0005524	ATP binding	48 (42/6)	1,06E-04
cellular_component	GO:0005737	cytoplasm	164 (120/44)	1,06E-04
molecular_function	GO:0003824	catalytic activity	129 (100/29)	1,20E-04
molecular_function	GO:0030554	adenyl nucleotide binding	50 (44/6)	1,25E-04
molecular_function	GO:0032559	adenyl ribonucleotide binding	48 (42/6)	1,40E-04
molecular_function	GO:0001883	purine nucleoside binding	50 (44/6)	1,54E-04
molecular_function	GO:0003924	GTPase activity	11 (9/2)	1,77E-04
molecular_function	GO:0001882	nucleoside binding	50 (44/6)	1,80E-04
biological_process	GO:0002684	positive regulation of immune system process	14 (9/5)	2,11E-04
molecular_function	GO:0016817	hydrolase activity, acting on acid anhydrides	27 (23/4)	3,59E-04
cellular_component	GO:0005622	intracellular	230 (165/65)	3,62E-04
biological_process	GO:0050776	regulation of immune response	13 (9/4)	3,83E-04
cellular_component	GO:0043231	intracellular membrane-bounded organelle	176 (127/49)	4,12E-04
cellular_component	GO:0043227	membrane-bounded organelle	176 (127/49)	4,33E-04
biological_process	GO:0006732	coenzyme metabolic process	12 (11/1)	5,05E-04
molecular_function	GO:0016462	pyrophosphatase activity	26 (22/4)	6,03E-04
biological_process	GO:0002682	regulation of immune system process	17 (12/5)	6,14E-04
molecular_function	GO:0016818	hydrolase activity, acting on acid anhydrides, in phosphorus-containing anhydrides	26 (22/4)	6,51E-04
molecular_function	GO:0017111	nucleoside-triphosphatase activity	25 (22/3)	7,69E-04
biological_process	GO:0002252	immune effector process	10 (7/3)	8,22E-04
cellular_component	GO:0043229	intracellular organelle	192 (137/55)	8,30E-04
cellular_component	GO:0043226	organelle	192 (137/55)	8,56E-04
biological_process	GO:0043603	cellular amide metabolic process	6 (6/0)	9,21E-04
molecular_function	GO:0005488	binding	241 (180/61)	9,58E-04
cellular_component	GO:0005739	mitochondrion	44 (30/14)	1,31E-03
biological_process	GO:0050778	positive regulation of immune response	10 (6/4)	1,41E-03
biological_process	GO:0002697	regulation of immune effector process	8 (5/3)	1,67E-03
biological_process	GO:0050870	positive regulation of T cell activation	7 (5/2)	1,80E-03
biological_process	GO:0048583	regulation of response to stimulus	16 (11/5)	2,16E-03
biological_process	GO:0044085	cellular component biogenesis	23 (12/11)	2,38E-03
biological_process	GO:0051251	positive regulation of lymphocyte activation	8 (6/2)	2,44E-03
biological_process	GO:0055086	nucleobase-containing small molecule metabolic process	14 (14/0)	2,50E-03
biological_process	GO:0050896	response to stimulus	65 (49/16)	2,84E-03
biological_process	GO:0051186	cofactor metabolic process	12 (11/1)	3,13E-03
cellular_component	GO:0009897	external side of plasma membrane	14 (9/5)	3,25E-03
biological_process	GO:0002696	positive regulation of leukocyte activation	8 (6/2)	3,26E-03
biological_process	GO:0019320	hexose catabolic process	6 (6/0)	3,38E-03
biological_process	GO:0006007	glucose catabolic process	6 (6/0)	3,38E-03
biological_process	GO:0009117	nucleotide metabolic process	13 (13/0)	3,47E-03
biological_process	GO:0006753	nucleoside phosphate metabolic process	13 (13/0)	3,47E-03
molecular_function	GO:0005525	GTP binding	16 (13/3)	3,51E-03
biological_process	GO:0050867	positive regulation of cell activation	8 (6/2)	3,65E-03
biological_process	GO:0046365	monosaccharide catabolic process	6 (6/0)	3,99E-03
molecular_function	GO:0019001	guanyl nucleotide binding	16 (13/3)	4,42E-03
molecular_function	GO:0032561	guanyl ribonucleotide binding	16 (13/3)	4,42E-03
biological_process	GO:0051704	multi-organism process	16 (13/3)	4,66E-03
biological_process	GO:0006950	response to stress	36 (26/10)	5,26E-03
biological_process	GO:0050863	regulation of T cell activation	8 (6/2)	5,27E-03
biological_process	GO:0045619	regulation of lymphocyte differentiation	6 (4/2)	5,42E-03
biological_process	GO:0044275	cellular carbohydrate catabolic process	6 (6/0)	6,27E-03
cellular_component	GO:0000502	proteasome complex	6 (6/0)	6,29E-03
biological_process	GO:0022607	cellular component assembly	19 (9/10)	6,54E-03
biological_process	GO:0009056	catabolic process	33 (26/7)	7,16E-03
biological_process	GO:0044237	cellular metabolic process	135 (105/30)	7,22E-03
biological_process	GO:0009987	cellular process	198 (139/59)	8,26E-03
cellular_component	GO:0005829	cytosol	21 (17/4)	8,46E-03
biological_process	GO:0046164	alcohol catabolic process	6 (6/0)	8,76E-03
biological_process	GO:0016043	cellular component organization	51 (30/21)	9,98E-03
biological_process	GO:0006096	glycolysis	5 (5/0)	1,05E-02
biological_process	GO:0048584	positive regulation of response to stimulus	10 (6/4)	1,10E-02
biological_process	GO:0006769	nicotinamide metabolic process	4 (4/0)	1,10E-02
biological_process	GO:0046496	nicotinamide nucleotide metabolic process	4 (4/0)	1,10E-02
biological_process	GO:0009820	alkaloid metabolic process	4 (4/0)	1,10E-02
biological_process	GO:0080134	regulation of response to stress	10 (7/3)	1,18E-02
molecular_function	GO:0016787	hydrolase activity	57 (42/15)	1,18E-02
biological_process	GO:0006954	inflammatory response	11 (7/4)	1,32E-02
biological_process	GO:0043933	macromolecular complex subunit organization	15 (6/9)	1,34E-02
biological_process	GO:0046635	positive regulation of alpha-beta T cell activation	4 (3/1)	1,38E-02
biological_process	GO:0045580	regulation of T cell differentiation	5 (3/2)	1,42E-02
biological_process	GO:0044248	cellular catabolic process	27 (21/6)	1,52E-02
biological_process	GO:0006957	complement activation, alternative pathway	3 (2/1)	1,55E-02
biological_process	GO:0019362	pyridine nucleotide metabolic process	4 (4/0)	1,69E-02
biological_process	GO:0051603	proteolysis involved in cellular protein catabolic process	19 (14/5)	1,77E-02
biological_process	GO:0044257	cellular protein catabolic process	19 (14/5)	1,86E-02
molecular_function	GO:0003723	RNA binding	22 (20/2)	1,90E-02
biological_process	GO:0030278	regulation of ossification	5 (5/0)	1,98E-02
biological_process	GO:0016053	organic acid biosynthetic process	8 (7/1)	2,09E-02
biological_process	GO:0046394	carboxylic acid biosynthetic process	8 (7/1)	2,09E-02

biological_process	GO:0006959	humoral immune response	5 (3/2)	2,11E-02
biological_process	GO:0016052	carbohydrate catabolic process	6 (6/0)	2,12E-02
biological_process	GO:0006633	fatty acid biosynthetic process	6 (5/1)	2,12E-02
cellular_component	GO:0005794	Golgi apparatus	23 (18/5)	2,14E-02
molecular_function	GO:0016616	oxidoreductase activity, acting on the CH-OH group of donors, NAD or NADP as acce	7 (7/0)	2,15E-02
biological_process	GO:0030641	regulation of cellular pH	3 (2/1)	2,21E-02
biological_process	GO:0006885	regulation of pH	4 (3/1)	2,22E-02
biological_process	GO:0051249	regulation of lymphocyte activation	8 (6/2)	2,31E-02
molecular_function	GO:0008134	transcription factor binding	12 (8/4)	2,33E-02
biological_process	GO:0032649	regulation of interferon-gamma production	4 (3/1)	2,41E-02
biological_process	GO:0045582	positive regulation of T cell differentiation	4 (3/1)	2,41E-02
biological_process	GO:0044238	primary metabolic process	138 (104/34)	2,52E-02
biological_process	GO:0030163	protein catabolic process	19 (14/5)	2,54E-02
biological_process	GO:0002711	positive regulation of T cell mediated immunity	3 (2/1)	2,58E-02
biological_process	GO:0002831	regulation of response to biotic stimulus	3 (2/1)	2,58E-02
biological_process	GO:0002253	activation of immune response	6 (4/2)	2,66E-02
biological_process	GO:0046634	regulation of alpha-beta T cell activation	4 (3/1)	2,83E-02
biological_process	GO:0045621	positive regulation of lymphocyte differentiation	4 (3/1)	2,83E-02
biological_process	GO:0031214	biomineral tissue development	4 (3/1)	3,05E-02
biological_process	GO:0015031	protein transport	21 (12/9)	3,09E-02
biological_process	GO:0006996	organelle organization	30 (19/11)	3,16E-02
biological_process	GO:0002694	regulation of leukocyte activation	8 (6/2)	3,18E-02
biological_process	GO:0002541	activation of plasma proteins involved in acute inflammatory response	4 (2/2)	3,28E-02
biological_process	GO:0006956	complement activation	4 (2/2)	3,28E-02
biological_process	GO:0001819	positive regulation of cytokine production	5 (3/2)	3,29E-02
biological_process	GO:0045184	establishment of protein localization	21 (12/9)	3,30E-02
molecular_function	GO:0016614	oxidoreductase activity, acting on CH-OH group of donors	7 (7/0)	3,32E-02
biological_process	GO:0050865	regulation of cell activation	8 (6/2)	3,37E-02
biological_process	GO:0051607	defense response to virus	3 (2/1)	3,38E-02
biological_process	GO:0065003	macromolecular complex assembly	13 (5/8)	3,45E-02
biological_process	GO:0016064	immunoglobulin mediated immune response	5 (4/1)	3,47E-02
biological_process	GO:0042127	regulation of cell proliferation	18 (10/8)	3,58E-02
molecular_function	GO:0008009	chemokine activity	4 (2/2)	3,58E-02
biological_process	GO:0048518	positive regulation of biological process	41 (28/13)	3,68E-02
biological_process	GO:0006733	oxidoreduction coenzyme metabolic process	4 (4/0)	3,77E-02
biological_process	GO:0055067	monovalent inorganic cation homeostasis	4 (3/1)	3,77E-02
molecular_function	GO:0004731	purine-nucleoside phosphorylase activity	2 (2/0)	3,79E-02
biological_process	GO:0070167	regulation of biomineral tissue development	3 (3/0)	3,82E-02
biological_process	GO:0030500	regulation of bone mineralization	3 (3/0)	3,82E-02
biological_process	GO:0019724	B cell mediated immunity	5 (4/1)	3,82E-02
biological_process	GO:0002706	regulation of lymphocyte mediated immunity	5 (4/1)	3,82E-02
molecular_function	GO:0042379	chemokine receptor binding	4 (2/2)	3,83E-02
biological_process	GO:0008104	protein localization	23 (13/10)	3,92E-02
biological_process	GO:0019941	modification-dependent protein catabolic process	17 (12/5)	4,22E-02
biological_process	GO:0043632	modification-dependent macromolecule catabolic process	17 (12/5)	4,22E-02
biological_process	GO:0032729	positive regulation of interferon-gamma production	3 (2/1)	4,27E-02
biological_process	GO:0030004	cellular monovalent inorganic cation homeostasis	3 (2/1)	4,27E-02
biological_process	GO:0002705	positive regulation of leukocyte mediated immunity	4 (3/1)	4,29E-02
biological_process	GO:0002708	positive regulation of lymphocyte mediated immunity	4 (3/1)	4,29E-02
biological_process	GO:0051246	regulation of protein metabolic process	13 (7/6)	4,36E-02
molecular_function	GO:0001664	G-protein coupled receptor binding	5 (3/2)	4,52E-02
molecular_function	GO:0042287	MHC protein binding	3 (3/0)	4,57E-02
biological_process	GO:0006605	protein targeting	7 (3/4)	4,65E-02
cellular_component	GO:0009986	cell surface	14 (9/5)	4,68E-02
biological_process	GO:0002709	regulation of T cell mediated immunity	3 (2/1)	4,74E-02
biological_process	GO:0002703	regulation of leukocyte mediated immunity	5 (4/1)	4,81E-02
cellular_component	GO:0005759	mitochondrial matrix	8 (5/3)	4,82E-02
cellular_component	GO:0030529	ribonucleoprotein complex	16 (11/5)	4,95E-02
biological_process	GO:0033036	macromolecule localization	26 (16/10)	4,97E-02
cellular_component	GO:0005761	mitochondrial ribosome	4 (2/2)	4,98E-02
cellular_component	GO:0000313	organellar ribosome	4 (2/2)	4,98E-02
biological_process	GO:0031347	regulation of defense response	6 (4/2)	5,15E-02
molecular_function	GO:0000287	magnesium ion binding	14 (12/2)	5,16E-02
molecular_function	GO:0005515	protein binding	128 (84/44)	5,21E-02
biological_process	GO:0046638	positive regulation of alpha-beta T cell differentiation	3 (2/1)	5,23E-02
biological_process	GO:0008284	positive regulation of cell proliferation	11 (5/6)	5,27E-02
biological_process	GO:0044265	cellular macromolecule catabolic process	19 (14/5)	5,35E-02
biological_process	GO:0008610	lipid biosynthetic process	11 (9/2)	5,38E-02
biological_process	GO:0008152	metabolic process	151 (113/38)	5,42E-02
biological_process	GO:0008283	cell proliferation	10 (7/3)	5,42E-02
molecular_function	GO:0070566	adenylyltransferase activity	3 (3/0)	5,54E-02
biological_process	GO:0001817	regulation of cytokine production	7 (5/2)	5,54E-02
biological_process	GO:0010888	negative regulation of lipid storage	2 (2/0)	5,75E-02
molecular_function	GO:0005125	cytokine activity	8 (2/6)	5,84E-02
cellular_component	GO:0005672	transcription factor TFIIA complex	2 (1/1)	5,95E-02
cellular_component	GO:0000139	Golgi membrane	7 (5/2)	6,01E-02
molecular_function	GO:0016740	transferase activity	43 (35/8)	6,04E-02
biological_process	GO:0002699	positive regulation of immune effector process	4 (3/1)	6,07E-02
biological_process	GO:0002449	lymphocyte mediated immunity	5 (4/1)	6,15E-02
biological_process	GO:0030217	T cell differentiation	5 (4/1)	6,15E-02
biological_process	GO:0045058	T cell selection	3 (2/1)	6,27E-02
biological_process	GO:0030258	lipid modification	4 (3/1)	6,39E-02
cellular_component	GO:0032993	protein-DNA complex	5 (0/5)	6,56E-02
biological_process	GO:0045321	leukocyte activation	9 (8/1)	6,64E-02
cellular_component	GO:0044429	mitochondrial part	17 (10/7)	6,74E-02
biological_process	GO:0046637	regulation of alpha-beta T cell differentiation	3 (2/1)	6,81E-02
molecular_function	GO:0016772	transferase activity, transferring phosphorus-containing groups	25 (19/6)	7,04E-02
biological_process	GO:0006091	generation of precursor metabolites and energy	10 (8/2)	7,12E-02
biological_process	GO:0044267	cellular protein metabolic process	50 (35/15)	7,16E-02
biological_process	GO:0030098	lymphocyte differentiation	6 (5/1)	7,29E-02
biological_process	GO:0009262	deoxyribonucleotide metabolic process	3 (3/0)	7,36E-02
biological_process	GO:0001912	positive regulation of leukocyte mediated cytotoxicity	3 (2/1)	7,36E-02

biological_process	GO:0006099	tricarboxylic acid cycle	3 (2/1)	7,36E-02
biological_process	GO:0031343	positive regulation of cell killing	3 (2/1)	7,36E-02
biological_process	GO:0001574	ganglioside biosynthetic process	2 (2/0)	7,59E-02
biological_process	GO:0050691	regulation of defense response to virus by host	2 (1/1)	7,59E-02
biological_process	GO:0032020	ISG15-protein conjugation	2 (2/0)	7,59E-02
biological_process	GO:0045595	regulation of cell differentiation	13 (10/3)	7,68E-02
biological_process	GO:0042110	T cell activation	6 (5/1)	7,73E-02
biological_process	GO:0070271	protein complex biogenesis	9 (5/4)	7,82E-02
biological_process	GO:0006461	protein complex assembly	9 (5/4)	7,82E-02
biological_process	GO:0046356	acetyl-CoA catabolic process	3 (2/1)	7,93E-02
biological_process	GO:0046128	purine ribonucleoside metabolic process	3 (3/0)	7,93E-02
biological_process	GO:0042278	purine nucleoside metabolic process	3 (3/0)	7,93E-02
biological_process	GO:0009611	response to wounding	12 (8/4)	7,94E-02
biological_process	GO:0007067	mitosis	7 (5/2)	8,01E-02
biological_process	GO:0000280	nuclear division	7 (5/2)	8,01E-02
molecular_function	GO:0016765	transferase activity, transferring alkyl or aryl (other than methyl) groups	5 (5/0)	8,10E-02
biological_process	GO:0046649	lymphocyte activation	8 (7/1)	8,17E-02
biological_process	GO:0002250	adaptive immune response	5 (4/1)	8,22E-02
biological_process	GO:0002460	adaptive immune response based on somatic recombination of immune receptors bui	5 (4/1)	8,22E-02
biological_process	GO:0051239	regulation of multicellular organismal process	22 (17/5)	8,39E-02
biological_process	GO:0008637	apoptotic mitochondrial changes	3 (3/0)	8,51E-02
biological_process	GO:0000087	M phase of mitotic cell cycle	7 (5/2)	8,70E-02
biological_process	GO:0048519	negative regulation of biological process	34 (22/12)	8,86E-02
biological_process	GO:0009057	macromolecule catabolic process	19 (14/5)	8,95E-02
biological_process	GO:0019882	antigen processing and presentation	5 (3/2)	9,08E-02
biological_process	GO:0044255	cellular lipid metabolic process	15 (12/3)	9,14E-02
cellular_component	GO:0044431	Golgi apparatus part	9 (6/3)	9,15E-02
biological_process	GO:0050793	regulation of developmental process	17 (13/4)	9,15E-02
molecular_function	GO:0032452	histone demethylase activity	2 (2/0)	9,22E-02
biological_process	GO:0048285	organelle fission	7 (5/2)	9,26E-02
biological_process	GO:0006886	intracellular protein transport	10 (6/4)	9,32E-02
biological_process	GO:0050688	regulation of defense response to virus	2 (1/1)	9,40E-02
biological_process	GO:0051279	regulation of release of sequestered calcium ion into cytosol	2 (2/0)	9,40E-02
cellular_component	GO:0005840	ribosome	8 (5/3)	9,63E-02
biological_process	GO:0002819	regulation of adaptive immune response	4 (3/1)	9,64E-02
biological_process	GO:0009116	nucleoside metabolic process	4 (4/0)	9,64E-02
biological_process	GO:0002822	regulation of adaptive immune response based on somatic recombination of immune	4 (3/1)	9,64E-02
biological_process	GO:0002443	leukocyte mediated immunity	5 (4/1)	9,67E-02
cellular_component	GO:0005615	extracellular space	16 (4/12)	9,69E-02
biological_process	GO:0009060	aerobic respiration	3 (2/1)	9,72E-02
biological_process	GO:0046456	icosanoid biosynthetic process	3 (2/1)	9,72E-02
cellular_component	GO:0031362	anchored to external side of plasma membrane	2 (2/0)	9,72E-02
cellular_component	GO:0046540	U4/U6 x U5 tri-snRNP complex	2 (1/1)	9,72E-02
biological_process	GO:0016485	protein processing	5 (2/3)	9,98E-02
